# A Novel Lipopeptide–Functionalized Metal–Organic Framework for Periodontitis Therapy through the Htra1/FAK/YAP Pathway

**DOI:** 10.34133/bmr.0057

**Published:** 2024-07-29

**Authors:** Xuechun Wang, Qing Wang, Jian Wang, Xuan Wang, Linling Yin, Changping Wang, Guangjian Fan, Jinsong Pan

**Affiliations:** ^1^ Department of Stomatology, Shanghai General Hospital, Shanghai Jiao Tong University School of Medicine, Shanghai, 201620, China.; ^2^ Department of General Dentistry, Shanghai Ninth People’s Hospital, School of Medicine, College of Stomatology, Shanghai Jiao Tong University, National Center for Stomatology, National Clinical Research Center for Oral Diseases, Shanghai Key Laboratory of Stomatology, Shanghai Research Institute of Stomatology, Research Unit of Oral and Maxillofacial Regenerative Medicine, Chinese Academy of Medical Sciences, Shanghai, 200011, China.; ^3^ Department of Orthopaedics, Shanghai General Hospital, Shanghai Jiao Tong University School of Medicine, Shanghai, 200080, China.; ^4^ Precision Research Center for Refractory Diseases, Institute for Clinical Research, Shanghai General Hospital, Shanghai Jiao Tong University School of Medicine, Shanghai, 200080, China.

## Abstract

Periodontitis is a chronic inflammatory disease characterized by plaque accumulation, resulting in immune microenvironment disorders and resorption of alveolar bone. To promote bone healing under inflammatory environments, a functional biomaterial based on disease pathophysiology is designed. A novel fatty acid C10-modified polypeptide, C_10_-KR8, is discovered to have excellent abilities in modulating macrophage repolarization and promoting bone regeneration in periodontitis. To build a multifunctional material localized drug delivery system, C_10_-KR8@ZIF-8 (C_10_-KR8-loaded zeolitic imidazolate framework-8) nanoparticles are constructed to sustainedly release the C_10_-KR8 peptide and Zn elements. By synergistic effects of providing a dynamic immuno-modulatory environment and promoting osteogenesis under pathological conditions, the obtained pH-responsive nanoparticles display excellent bone regeneration capability. Furthermore, coimmunoprecipitation/liquid chromatography-tandem mass spectrometry analysis and proteomics analysis revealed that the C_10_-KR8 peptide directly interacts with the high-temperature requirement protein A1 (Htra1), and C_10_-KR8@ZIF-8 nanoparticles promote the osteogenic differentiation of bone mesenchymal stem cells by activating the focal adhesion kinase (FAK)/phosphatidylinositide 3-kinase (PI3K)/AKT pathway and enhancing the nuclear localization of Yes-associated protein (YAP). Taken together, this study demonstrates C_10_-KR8 peptide regulate osteoimmunology and bone regeneration by Htra1/FAK/YAP pathway and that ZIF-8-based peptide loading platform is a promising strategy for periodontitis.

## Introduction

Periodontitis is a common and destructive inflammatory disease initiated by the dental plaque that progressively causes gingivitis and destroys the supporting structures such as the cementum and alveolar bone, eventually leading to tooth loss [1]. There is a link between periodontitis and systemic diseases, such as cardiovascular diseases, insulin resistance, diabetes, and colorectal cancer [2]. At present, the primary approach for treating periodontitis involves the use of mechanical cleaning, antibiotics, and surgical methods [3]. Additionally, immunomodulation can be employed to prevent the progression of persistent inflammation [1]. The inflammatory microenvironment at the local level increases susceptibility to dysfunction of functional cells such as macrophages and osteoblasts, leading to hindered bone regeneration and limited effectiveness of current clinical strategies [4]. Furthermore, the loss of alveolar bone is a substantial and permanent indication of periodontal disease, and current treatment methods have had limited effectiveness in promoting natural in periodontitis [5]. Consequently, the transition from an exacerbated inflammatory microenvironment to one that promotes bone regeneration in periodontitis patients remains a formidable obstacle.

Antimicrobial peptides (AMPs) have emerged as new and noteworthy candidates for periodontitis treatment due to their antibiotic properties and immune-regulating functions during inflammatory processes [6,7]. Among these AMPs, LL-37 has been shown to demonstrate broad bactericidal activity against both gram-positive and gram-negative bacteria [8] and to have other bioactivities, including the ability to neutralize lipopolysaccharides (LPSs), modulate the inflammatory response [9], promote wound healing[10], and facilitate the osteogenesis of mesenchymal stem cells (MSCs) [11,12]. However, the use of long peptides presents a challenge due to the associated high production costs, thereby restricting their clinical application. Upon analyzing a restricted selection of short peptides that encompass the complete LL-37 sequence [13], we discovered that KR8, which features the smallest molecular unit of LL-37, efficiently retains its activity [14].

A range of peptides are frequently modified by methods such as the inclusion of modified amino acids, cyclization, lipidation, and glycosylation. Fatty acid modification can improve the solubility and absorption of peptides, inhibit the degradation of proteases, and increase the binding rate of plasma albumin [15]. Daptomycin and colistin are 2 peptides that are currently used as vital antibiotics in clinical practice [15,16]. It has been observed that KR8, when conjugated with C10 fatty acids, exhibits outstanding antibacterial properties [17]. However, less attention has been given to its potential role in modulating the immune response and promoting osteogenesis in MSCs. Furthermore, the effectiveness of C_10_-KR8 may be limited due to its susceptibility to proteolysis [17]. Consequently, a microenvironment-responsive biomaterial that can regulate the controlled release of C_10_-KR8 and maintain its functional concentration is needed.

MOFs, also known as metal–organic frameworks, are a highly promising group of solid-state materials with potential in multiple areas including gas storage [18], catalysis, sensing, and imaging [19]. These unique properties make them an ideal candidate for carrying C_10_-KR8. Zeolitic imidazolate framework-8 (ZIF-8), as a type of MOF, has attracted considerable interest due to its impressive porosity, extensive surface area, and favorable biocompatibility [20]. Multiple research studies have shown that ZIF-8 provides a convenient framework for linking small compounds, peptides, enzymes, and proteins because of its gentle and uncomplicated synthesis circumstances [21,22]. In particular, ZIF-8 has been reported to exhibit sustained release of Zn element, which is known for its effective role in osteogenesis [23,24], anti-inflammatory activity [25], and resistance to pathogenic microorganisms [26,27]. Moreover, ZIF-8 is pH responsive, which is beneficial for delivering drugs under acidic conditions [28]. The presence of bacterial infections can lead to the accumulation of acidic metabolites, resulting in the creation of an acidic milieu. This approach offers a promising opportunity for the targeting of pH-sensitive carriers [22]. To summarize, ZIF-8 is an exceptional framework for delivering peptides because of its remarkable attributes of high aqueous stability and porosity, making it a superb option for medical biomaterials, especially in the realm of periodontitis.

**Fig. 1.**
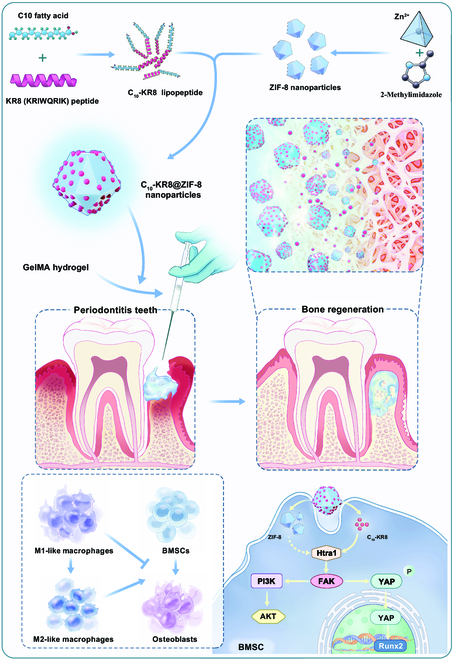
Schematic diagram of this research. A pH-responsive ZIF-8-based peptide loading platform was synthesized. The C_10_-KR8@ZIF-8 nanoparticles could release lipopeptide C_10_-KR8 and Zn ions, regulating macrophages polarization, interacting with Htra1 protein, or activating Htra1/FAK/YAP pathway to promote osteogenesis in vitro and in vitro*.*

In our study, we conjugated a newly synthesized peptide, KR8 (KRIWQRIK) with C10 fatty acids and investigated its anti-inflammatory properties, ability to modulate macrophage polarization, and potential role in promoting the osteogenesis of bone marrow-derived mesenchymal stem cells (BMSCs) under inflammatory conditions. Furthermore, we developed a peptide-functionalized ZIF-8 nanodrug delivery system to achieve a sustained release of C_10_-KR8 over an extended period of time. Moreover, we developed gelatin-methacryloyl (GelMA) hydrogels that can be injected with C_10_-KR8@ZIF-8 nanoparticles to study the effectiveness of C_10_-KR8@ZIF-8 in the treatment of inflammation-induced bone loss in a rat periodontitis model. Finally, we conducted an experimental analysis to characterize the osteogenic role of C_10_-KR8@ZIF-8 by utilizing coimmunoprecipitation (Co-IP)/liquid chromatography-tandem mass spectrometry (LC-MS/MS). This compound attaches to the high-temperature requirement protein A1 (Htra1) subunit, enhances the nuclear localization of Yes-associated protein (YAP), and triggers the activation of focal adhesion kinase (FAK)/phosphatidylinositide 3-kinase (PI3K)/AKT signaling. This study aimed to develop a novel peptide-modified MOF nanoparticle as a potential candidate for the treatment of periodontitis. Figure 1 shows the main intention of the present study.

## Materials and Methods

### Materials

The C_10_-KR8 peptide (C_10_-KRIWQRIK) was synthesized using the solid-phase technique and purified to a purity level exceeding 95% (NJPeptide, Nanjing, China). LPS (Sigma, USA) was extracted from *Escherichia coli* (*E. coli*) and dissolved in phosphate-buffered saline (PBS). PBS was obtained from Gibco (USA). Gibco (USA) supplied Dulbecco’s modified Eagle’s medium (DMEM), alpha minimal essential medium (α-MEM), fetal bovine serum (FBS), and penicillin–streptomycin (PS).

### Cell culture

BMSCs derived from the femurs and tibias of 3-week-old Sprague Dawley rats were extracted and placed in a 5% CO_2_ incubator at 37 °C. The cells were then cultured in 𝛼-MEM supplemented with 10% FBS and 1% PS. The cells were passaged at approximately 80% confluence and used at early passages (p3–5). Next, the medium s𝛼-MEM supplemented with 10% FBS, 1% PS, 1 mM dexamethasone, 10 mM l-ascorbic acid, and 1 M 𝛽-glycerophosphate.

Murine macrophage RAW 264.7 cells were purchased from the China Center for Type Culture Collection . The cells were grown in DMEM supplemented with 10% FBS and 1% PS and placed in a 5% CO_2_ incubator at 37 °C.

Bone marrow-derived macrophages (BMDMs) were acquired from the tibia and femurs of C57BL/6 mice aged between 8 and 12 weeks. The cells were grown in DMEM supplemented with 10% (v/v) FBS and 1% PS. Afterward, the cells were treated with 25 ng/ml mouse M-CSF (BioLegend, USA) for a period of 7 d to induce differentiation.

### Collection of conditioned medium

The macrophage conditioned medium (CM) was collected following the procedure outlined in a previous publication [29]. In short, macrophages were cultured with C_10_-KR8 for 24 h, followed by incubation with or without LPS (100 ng/ml) for another 24 h. Afterward, the collected medium was centrifuged at a speed of 14,000 rpm for 30 min. Next, the obtained supernatant was combined with regular osteogenic medium at a 1:2 ratio to generate the CM for subsequent experiments.

### Quantitative real-time PCR

Following the manufacturer’s instructions, TRIzol reagent (TaKaRa, Japan) was used to extract total RNA from the cultured cells. The PrimeScript RT Reagent Kit (TaKaRa, Japan) was used to generate complementary DNA from the isolated RNA. A NanoDrop spectrophotometer was used to measure the concentration and purity of the bacteria. Following the reverse transcriptionpolymerase chain reaction (RT-PCR), a TB Green Premix Ex Taq Reagent Kit (TaKaRa, Japan) and an ABI Real-Time PCR system (Life Technologies, USA) were utilized for real-time PCR. The expression data were standardized to the glyceraldehyde-3-phosphate dehydrogenase (GAPDH) data to manage the variation in expression levels. Relative quantification was determined as the comparative 2−ΔΔCt method, and each experiment was replicated 3 times. The primers used are listed in Table S1.

### Western blot analysis

After the cells were cultured for different durations, the cells were washed with PBS and lysed with radioimmunoprecipitation assay lysis buffer (Beyotime, China). The cell lysates were centrifuged at 4 °C and 12,000 rpm for 15 min. The bicinchoninic acid method (Beyotime) was used to analyze the protein concentration. Sodium dodecyl sulfate–polyacrylamide gel electrophoresis (GenScript, China) was used to separate the proteins, which were subsequently transferred to nitrocellulose membranes (Bio-Rad, USA). After blocking in 5% skim milk for 1 h (Beyotime), the membrane was subsequently incubated with primary antibodies (Table S2) overnight at 4 °C. Afterward, the samples were incubated with a secondary antibody (Table S3) for 1 h, and the signals were visualized with a Tanon detection system (Tanon Science & Technology Co., China).

### Synthesis of ZIF-8 and C_10_-KR8@ZIF-8

In short, a solution of Zn (NO_3_)_2_·6H_2_O (500 mg) was prepared by dissolving it in 5 ml of double-distilled H_2_O. Deionized water (10 ml) was used to disperse 2-mIM (1.15 g). The 2 solutions were mixed until transparent and then promptly combined at room temperature. After 5 min of magnetic stirring, the mixture was centrifuged (12 000 rpm for 15 min), after which the white solid products were collected and subsequently washed several times with MeOH. The remaining solvent was then removed under vacuum. The C_10_-KR8@ZIF-8 MOFs were prepared using a procedure identical to that used for ZIF-8, except that a double-distilled H_2_O solution (10 ml) was used to dissolve 2-mIM (1.15 g) and C_10_-KR8 (30 mg). The solutions were combined and allowed to react for 1 h. The solutions were mixed and allowed to react for another 2 h. The drying procedure was the same as that previously described.

### Characterization of the nanoparticles

The morphology of the nanoparticles was detected using a scanning electron microscope (SEM) (S4800, Hitachi, Japan) and transmission electron microscopy (TEM) (HT7800, Hitachi, Japan). One hundred nanoparticles were randomly selected from the images, and the diameter was calculated by ImageJ. To determine the phase composition of the nanoparticles, the ZIF nanoparticles were examined and studied using x-ray diffraction (XRD) equipment with Cu Kα radiation (ƛ = 1.541874) operating at 40 kV and 40 mA (D8, Germany). Information was gathered for 2𝜃 scanned from 10° to 70°. The scanning rate was set at 0.5°/min. To examine the group structure of the different nanoparticles, Fourier transform infrared (FT-IR) spectroscopy was used to analyze the nanoparticles in the wavelength range of 4,000 to 400 cm^−1^ (PerkinElmer Spectrum RX1 System).

### Co-IP and LC-MS/MS

The FLAG peptide-labeled C_10_-KR8 (C10-KRIWQRIK-DYKDDDDK) was constructed by NJPeptide (Nanjing, China). BMSCs were grown in cell culture dishes until they reached 80% confluence. Next, the dishes were incubated with 300 μg of C_10_-KR8-FLAG for 4 h. One milliliter of IP lysis buffer (Beyotime, China) was used to extract the total proteins. Approximately 200 μg of total protein was extracted from the cells and then incubated overnight at 4 °C with gentle rotation using anti-Flag magnetic beads (Bimake, USA). Subsequently, the peptide–protein complexes were extracted using magnetic separation. After the samples were boiled at 95 °C for 10 min, they were separated via sodium dodecyl sulfate–polyacrylamide gel electrophoresis. Subsequently, a Coomassie blue staining kit (Beyotime, China) was used. BiotechPack Scientific company conducted the complete LC-MS/MS process. The Western blot procedures used are described above.

### Plasmid construction and transfection

Small interfering RNA (siRNA) sequences targeting Htra1 (siHtra1) and a negative control (NC) were synthesized by GenePharma Company (Shanghai, China) (Table S1). The overexpression plasmid construct GV492-3FLAG-Htra1 was created and obtained from Gikai Gene Company (Shanghai, China). Transfection was performed using the Lipofectamine 3000 Transfection Kit (Invitrogen, L3000-015). The transfection process was carried out in accordance with the guidelines provided by the manufacturer.

### MS proteomics and data analysis of the BMSCs

BMSCs were treated with C_10_-KR8@ZIF-8 and transfected with NC siRNA or Htra1 siRNA. Following enzymatic hydrolysis, the nonlabeled quantitative (label-free) method was utilized to perform MS analysis of the enzymatic peptide fragments derived from the proteins. Peptide signals were identified by an LC-MS/MS system during the differentially expressed protein analysis. Pathway enrichment between the groups was analyzed using Gene Ontology (GO) and Kyoto Encyclopedia of Genes and Genomes (KEGG) enrichment analyses. The protein–protein interaction (PPI) network was constructed with the GeneMANIA database.

### In vivo study

The Ethics Committee of Shanghai General Hospital (ethics approval number 2021AW005) granted approval for all animal experiments. To evaluate the overall therapeutic impact of C_10_-KR8@ZIF-8 on periodontitis, a rat model of typical periodontitis induced by ligature was created. After 1 week of acclimatization, a total of 25 male Sprague–Dawley rats (6 weeks of age and weighing between 150 and 200 g) were randomly assigned to 5 different groups, and the rats were anesthetized by intraperitoneal injection of sodium pentobarbital (Merck Millipore, Germany). To induce periodontitis in the rats, a 4-0 ligature was tied around the bilateral maxillary first molars (M1) once a day. After a period of 4 weeks, GelMA hydrogels containing C_10_-KR8, ZIF-8, or C_10_-KR8@ZIF-8 (or a control solution of 0.9% NaCl) were injected into the periodontal pockets twice a week. Four weeks later, all the rats were euthanized using an excess amount of anesthesia. Samples were collected for quantitative RT-PCR (qRT-PCR) analysis, microcomputed tomography (micro-CT) examination, hematoxylin-eosin (H&E) staining, Masson staining, and immunofluorescence (IF) staining. The experimental details can be found in the Supplementary Materials.

### Tissue RNA extraction and qRT-PCR analysis

After a 4-week treatment with PBS or different GelMA groups, the Sprague Dawley rats with periodontitis were euthanized. The alveolar bone tissue surrounding the maxillary first molar was collected and washed with precooled normal saline to minimize the presence of red blood cells. Subsequently, the tissue was promptly frozen in liquid nitrogen and ground into powder. TRIzol reagent (TaKaRa, Japan) was used to extract total RNA from the periodontal tissues. The PrimeScript RT Reagent Kit (TaKaRa, Japan) was used to generate complementary DNA from the isolated RNA. Following the RT reaction, a TB Green Premix Ex Taq Reagent Kit (TaKaRa, Japan) and ABI Real-Time PCR system (Life Technologies, USA) were utilized for the execution of real-time PCR. Relative quantification was determined as the comparative 2−ΔΔCt. Runx2, osteopontin (OPN), interleukin-1β (IL-1β), IL-6, IL-4, and transforming growth factor-β (TGF-β) mRNA expression were evaluated, and GAPDH was used as the control gene. The primer sequences are shown in Table S4.

### Statistical analysis

The experiments were independently conducted at least 3 times (*n* ≥ 3). The data are presented as the mean ± standard deviation (SD). The figure legends specify the data analyzed with normalization. Differences among the groups were analyzed using Student *t* test or one-way analysis of variance, followed by Tukey’s post hoc test. Differences for which *P < 0.05, **P < 0.01, and ***P < 0.001 were considered to indicate statistically significant differences. All the statistical analyses were performed using GraphPad Prism 8.0 software.

## Results

### Effects of C_10_-KR8 on the regulation of inflammation and macrophage polarization

**Fig. 2.**
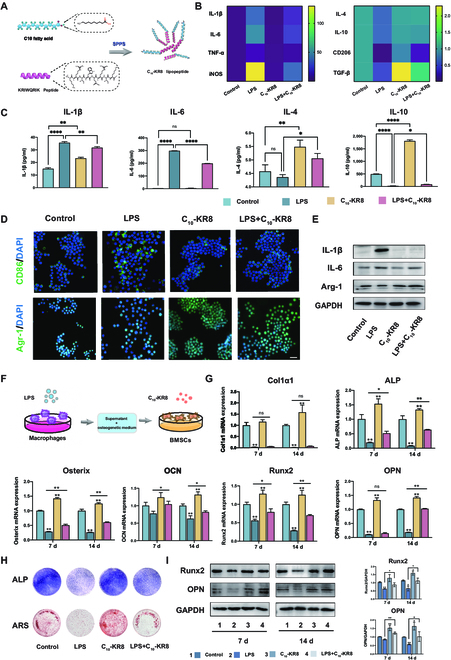
C_10_-KR8 regulate macrophage polarization and modulate crosstalk between macrophages and BMSCs. (A) Schematic representation of the KR8 peptide (KRIWQRIK) and C10 fatty acid. (B) The gene expression of the inflammation-related cytokines were quantified in RAW 264.7 cells via qRT-PCR. (C) ELISA was used to measure the levels of inflammation-related cytokines in the supernatant of RAW 264.7 cells. (D) Representative IF images of CD86+ and Arg-1+ cells among RAW 264.7 cells. Scale bar = 100 μm. (E) The levels of proteins associated with macrophage polarization were assessed in RAW264.7 cells via Western blot analysis. (F) An illustration in the form of a diagram. (G) ALP staining on day 7 and ARS staining images on day 14 of the BMSCs cultured in the respective CM. (H) The mRNA expression levels of the osteogenic genes in BMSCs were measured after 7 and 14 d of stimulation with various types of CM. (I) Western blot analysis of the relative protein expression levels of Runx2 and OPN in BMSCs at 7 and 14 d. The data are presented as the mean ± SD (*n* = 3). ns, not significant; *P < 0.05, **P < 0.01, ***P < 0.001, and ****P < 0.0001. DAPI, 4′,6-diamidino-2-phenylindole.

In this study, we utilized the midchain fatty acid C10 to modify the peptide KR8 and produced the peptide C_10_-KR8 (C10-KRIWQRIK) by a solid-phase method (Fig. 2A). The analysis conducted using high-performance liquid chromatography indicated that the purity exceeded 95%. To evaluate the biocompatibility of C_10_-KR8 in RAW264.7 cells, the Cell Counting Kit-8 (CCK-8) assay was used. During culture, RAW264.7 cells exhibited a typical growth pattern, and no apparent cytotoxicity was observed across the concentration range of 12.5 to 50 μg/ml for C_10_-KR8. The OD values of the 12.5 and 25 μg/ml groups were significantly greater than those of the 0 and 50 μg/ml groups at both 24 and 48 h (Fig. S1A).

Under periodontitis conditions, the macrophage has difficulties in shifting from an inflammatory phenotype (M1) to an anti-inflammatory phenotype (M2) due to the inflammation microenvironment, which has close connection with bone loss. To investigate the anti-inflammatory properties of C_10_-KR8 through immunomodulation, we used LPS to induce proinflammatory conditions. RAW264.7 cells were treated with various concentrations of C_10_-KR8, and the qRT-PCR findings indicated that C_10_-KR8 reversed the increase in the expression of the proinflammatory cytokines IL-1β, IL-6, tumor necrosis factor-α (TNF-a), and inducible nitric oxide synthase (iNOS) induced by LPS stimulation at 12.5 to 25 μg/ml. In contrast, compared with those in the LPS group, the IL-4 and IL-10 levels in the LPS+C_10_-KR8 group were significantly greater, and the TGF-β level in the C_10_-KR8 group was even greater than that in the control group (Fig. 2B and Fig. S1B). However, under stimulation of 50 μg/ml C_10_-KR8, neither inflammatory nor anti-inflammatory cytokines were down-regulated (Fig. S1B). This may be because the concentration of C_10_-KR8 was too high to exert normal effects. Considering its outstanding biocompatibility and ability to reduce inflammation in macrophages, it was concluded that a concentration of 25 μg/ml is the most suitable concentration for C_10_-KR8 treatment. The enzyme-linked immunosorbent assay (ELISA) results for IL-1β, IL-6, IL-4, and IL-10 aligned with the qRT-PCR findings, providing additional evidence that C_10_-KR8 has the capacity to reduce the expression of inflammatory substances and enhance the secretion of anti-inflammatory substances (Fig. 2C).

To explore whether C_10_-KR8 regulates the transition of macrophages from M1 to M2 phenotype, we examined the expression of CD86, a marker of M1 macrophages, as well as the M2-specific markers CD206 and arginase-1 (Arg-1). IF staining and flow cytometry analysis revealed an increase in the ratio of CD206- and Arg-1-positive M2 macrophages, whereas the expression level of CD86 in RAW264.7 cells decreased when C_10_-KR8 was added under inflammatory conditions (Fig. 2D and Fig. S1C and D). Moreover, the Western blot results further verified the ability of C_10_-KR8 to reprogram cells (Fig. 2E and Fig. S1E and F). Taken together, these results suggested that C_10_-KR8 effectively reduced the inflammatory response through polarizing macrophages to an anti-inflammatory phenotype.

### C_10_-KR8 enhances the osteogenesis through modulating crosstalk between macrophage and BMSCs

To further examine the impact of C_10_-KR8 on the osteogenic differentiation of BMSCs in CM from various macrophages, RAW264.7 cells were preincubated with C_10_-KR8 and/or LPS for 24 h. Then, the supernatants were collected and cultured with BMSCs to analyze osteogenic differentiation under various conditions (Fig. S2A).

Alkaline phosphatase (ALP), a signature enzyme of mature osteoblasts, was used to detect the osteogenic differentiation of the BMSCs via ALP staining. Moreover, as calcium salt deposition and bone matrix mineralization are crucial stages in bone formation, an Alizarin Red S (ARS) staining solution was used to identify mineralized calcium nodules, which serve as a late marker of calcium deposition. The findings suggested that under conditions of LPS-stimulated inflammation, the osteogenic differentiation of BMSCs was inhibited, and C_10_-KR8-mediated regulation of the inflammatory environment enhanced ALP expression, but it did not augment the development of calcium nodules (Fig. S2B). Similarly, the mRNA expression levels of Col1a1, Runx2, and OPN were greater in BMSCs cultured in C_10_-KR8-modulated inflammatory macrophage CM (LPS + C_10_-KR8 group) than in those cultured in the LPS group on day 7. Notably, in a noninflammatory environment, CM produced by C_10_-KR8 could increase Col1a1, Runx2, OPN, and OCN expression on day 14 better than the control group. This maybe because C_10_-KR8-induced macrophages release osteoinductive cytokines like TGF-β (Fig. S2C). Protein expression followed a similar trend, Western blot analysis revealed an increase in the protein expression of Runx2 and OPN in the LPS+C_10_-KR8 group compared to the LPS group on day 14. (Fig. S2D and E).

In addition to regulating the LPS-stimulated inflammatory microenvironment to enhance the osteogenic differentiation of BMSCs, C_10_-KR8 could directly promote the osteogenesis of BMSCs by activating them. We used different concentrations of C_10_-KR8 to treat BMSCs to evaluate the optimal concentration range of the lipopeptide and found that 20 μg/ml C_10_-KR8 exerts an outstanding up-regulation of genes related to osteogenesis (Fig. S3). To simulate inflammatory conditions in periodontitis, we cultured BMSCs in the CM of LPS-induced Raw264.7 cells. C_10_-KR8 was added to BMSCs to investigate its influence on osteogenic differentiation (Fig. 2F). In the inflammatory microenvironment, C_10_-KR8 directly modulated BMSCs, leading to increased gene expression levels of ALP, Runx2, OCN, and Osterix on day 7 compared to those in the LPS group (Fig. 2G). Furthermore, the application of C_10_-KR8 to BMSCs enhanced ALP activity and the formation of calcium nodules in an inflammatory environment (Fig. 2H). Notably, in the absence of LPS stimulation, the BMSCs treated with C_10_-KR8 alone exhibited better osteogenic differentiation than did those in the control group. Therefore, C_10_-KR8 can potentially enhance the osteogenic potential of BMSCs by directly stimulating BMSCs in the CM of macrophages in inflammatory or noninflammatory environments. Western blot analysis was also conducted to validate this hypothesis (Fig. 2I).

By effectively modulating the interaction between macrophages and BMSCs, C_10_-KR8 promoted osteogenic differentiation through its combined regulatory influence on both cell types (Fig. S4A). Figure S4B showed the ALP and ARS staining results, as the group that received the combination treatment showed a notably greater intensity of ALP staining than the LPS group. Importantly, more mineral nodules generated by BMSCs in the C_10_-KR8-modulated inflammatory environment than in the LPS group were observed through ARS staining (Fig. S4B). qRT-PCR results showed that C_10_-KR8 strongly enhanced the ALP, Runx2, OCN, OPN, and Osterix mRNA expression levels (Fig. S4C). Moreover, it increased the protein expression levels of Runx2 and OPN in both noninflammatory and inflammatory situations (Fig. S4D and E). Collectively, these results demonstrated that C_10_-KR8 can efficiently enhance the process of osteogenesis by regulating the communication between macrophages and BMSCs. These findings indicated that C_10_-KR8 has significant value and potential for regulating osteoimmunology and bone regeneration in periodontitis.

### Composition and characterization of C_10_-KR8@ZIF-8

We have demonstrated the bone immunomodulatory and osteogenic properties of C_10_-KR8 in previous experiments. However, considering the C_10_-KR8 exhibits limited stability and a short half-life due to enzymatic degradation, ZIF-8 has been chosen to be a carrier to load and realize long-term release of C_10_-KR8 peptide.

**Fig. 3.**
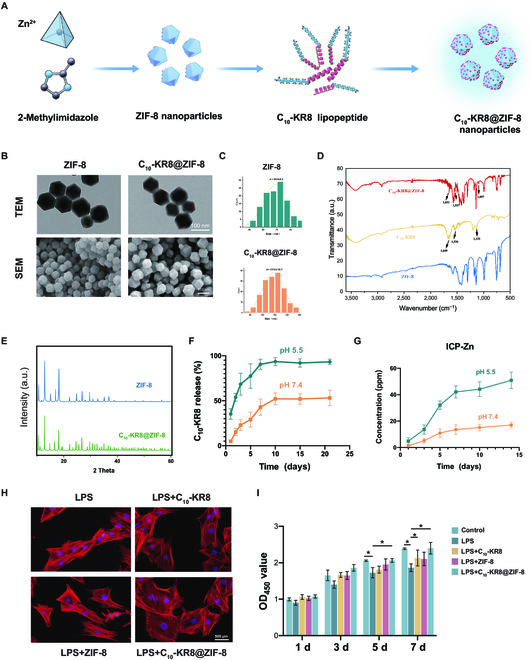
Composition and characterization of C_10_-KR8@ZIF-8. (A) Preparation scheme of ZIF-8, and C_10_-KR8@ZIF-8. (B) TEM and SEM images of ZIF-8 and C_10_-KR8@ZIF-8. (C) Size measurements of the nanoparticles. *n* =100. (D) FT-IR spectra of C_10_-KR8, ZIF-8, and C_10_-KR8@ZIF-8. (E) XRD patterns of ZIF-8 and C_10_-KR8. (F) Cumulative release of C_10_-KR8 from C_10_-KR8@ZIF-8. (G) Cumulative release of Zn^2+^ from C_10_-KR8@ZIF-8. (H) The morphology of BMSCs grown with various types of nanoparticles was evaluated through staining with phalloidin and DAPI. (I) BMSCs proliferation was detected by a CCK-8 assay on days 1, 3, 5, and 7. The data are presented as the mean ± SD (*n* = 3). *P < 0.05 compared with the control group. OD_450_, optical density at 450 nm.

The design and preparation of ZIF-8 and the novel multifunctional synergistic nanoparticles C_10_-KR8@ZIF-8 were synthesized by the one-pot method (Fig. 3A). Monodisperse polyhedral nanoparticles of ZIF-8 and C_10_-KR8@ZIF-8 were observed via TEM and scanning electron microscopy (SEM), as shown in Fig. 3B. The particle size of ZIF-8 was measured to be 68.3 ± 8.3 nm, whereas the size of the C_10_-KR8@ZIF-8 nanoparticles was greater, at 117.6 ± 16.1 nm (Fig. 3B and C). The FT-IR spectra exhibited unique crystal peaks corresponding to both C_10_-KR8 and ZIF-8 in the C_10_-KR8@ZIF-8 group (Fig. 3D), suggesting successful loading of C_10_-KR8 and a relatively stable structure. Moreover, The XRD patterns exhibited characteristic diffraction peaks typical of the ZIF-8 structure, indicating a high degree of crystallinity in the C_10_-KR8@ZIF-8 samples (Fig. 3E). Energy-dispersive spectroscopy (EDS) analyses (Fig. S5A) verified the presence of the C_10_-KR8 peptide in the C_10_-KR8@ZIF-8 nanoparticles, which was consistent with the elemental mapping analysis (Fig. S5B). These findings confirmed that we successfully synthesized ZIF-8 and C_10_-KR8@ZIF-8 nanoparticles with regular and uniform morphologies.

We employed bicinchoninic acid analysis to detect the release of the C_10_-KR8 peptide (Fig. 3F). The data indicated an initial rapid release of the drug before day 5, followed by a sustained decrease in release over time. Additionally, inductively coupled plasma optical emission spectrometry was utilized to examine the total liberation of Zn ions from ZIF-8 and C_10_-KR8@ZIF-8 (Fig. 3G). The results revealed the rapid release of Zn ions during the initial 5 d, followed by gradual and sustained release from C_10_-KR8@ZIF-8. To further investigate the differential release behavior of Zn ions and C_10_-KR8 under varying pH conditions, the materials were exposed to an acidic solution (pH = 5.5) to simulate a plaque environment. In the acidic environment, ZIF nanoparticles exhibited a greater rate of release of both Zn ions and the C_10_-KR8 peptide than did those in the neutral environment (Fig. 3F and G). This observation suggested that the release of C_10_-KR8 from ZIF-8 primarily occurred via a diffusion-controlled mechanism. Furthermore, these findings indicated that C_10_-KR8@ZIF-8 nanoparticles can be used for long-term C_10_-KR8 delivery, which is advantageous for facilitating in vivo damage repair.

To confirm the impact of C_10_-KR8@ZIF-8 on cellular structure during inflammation, we treated BMSCs with CM from LPS-stimulated mouse BMDMs to create an inflammatory environment. Figure 3H depicts the shrinkage of cells upon culture in the inflammatory microenvironment. However, in the C_10_-KR8@ZIF-8 group, the BMSCs exhibited an elongated cell shape and mitigated the detrimental effects of inflammation on cell adhesion. The proliferation of BMDMs and BMSCs treated with gradient concentrations of ZIF-8 and C_10_-KR8@ZIF-8 was evaluated using a CCK-8 assay (Fig. 3I and Fig. S5C and D). Compared with those in the control group, the cells in the inflammatory environment exhibited decreased growth on days 5 and 7. In contrast, the cells cultured with C_10_-KR8@ZIF-8 exhibited significant growth enhancement on day 7 compared to that of the LPS group. Moreover, live/dead staining demonstrated that C_10_-KR8, ZIF-8, and C_10_-KR8@ZIF-8 did not cause noticeable toxicity to BMSCs or macrophages (Fig. S5E). Collectively, these results validated the successful construction of a peptide delivery platform for future experiments.

### Effects of C_10_-KR8@ZIF-8 nanoparticles on inflammation through immunomodulation

**Fig. 4.**
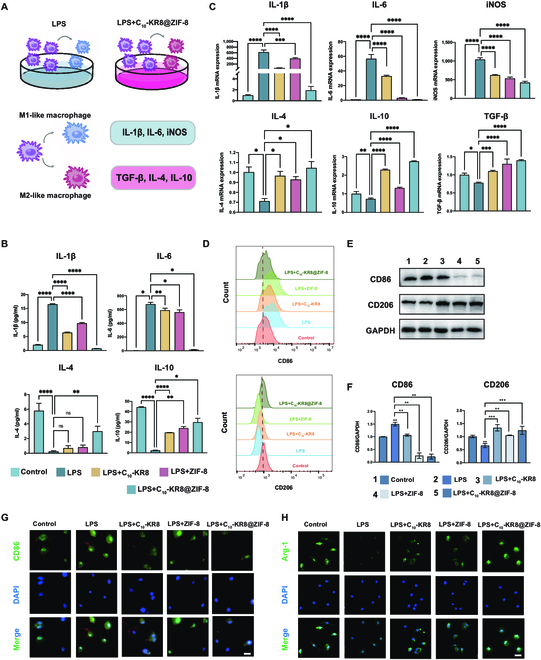
Effects of C_10_-KR8@ZIF-8 nanoparticles on inflammation through immunomodulation. (A) An illustration in the form of a diagram. (B) Gene expression of the inflammation-related cytokines in BMDMs was measured by qRT-PCR. (C) Concentrations of inflammation-related cytokines in the BMDM cell supernatant were measured via ELISA. (D) Flow cytometry analysis of CD86 and CD206 expression in BMDMs. (E and F) Western blot analysis of the expression of macrophage polarization-related proteins in BMDMs. (G and H) Representative IF images of CD86+ and Arg-1+ cells among BMDMs. Scale bar = 50 μm. The data are presented as the mean ± SD (*n* = 3). ns, not significant; *P < 0.05, **P < 0.01, ***P < 0.001, and ****P < 0.0001.

We previously demonstrated the anti-inflammatory function of C_10_-KR8. To further investigate the impact of the composite nanoparticles on inflammation through immunomodulation, BMDMs were cultured and identified via positive expression of the macrophage marker F4/80 by flow cytometry (Fig. S5F). To simulate an irritated microenvironment, BMDMs were prestimulated with LPS (100 ng/ml) for 24 h. After treatment with the nanoparticles for another 24 h, the macrophage polarization of the BMDMs was examined (Fig. 4A).

The qRT-PCR and ELISA results provided precise confirmation of the anti-inflammatory and reprograming ability of C_10_-KR8@ZIF-8. When BMDMs were stimulated with LPS and cultured with C_10_-KR8@ZIF-8, the expression of M1 phenotype markers and inflammatory markers (IL-1β, IL-6, and iNOS) decreased, whereas the expression of M2 phenotype markers and inflammatory markers (IL-4, IL-10, and TGF-β) significantly increased even further than that in the C_10_-KR8 group (Fig. 4B and C).

Flow cytometry and Western blot analysis and were conducted to detect polarization of macrophages. Expression of CD86 was greatly decreased in LPS+C_10_-KR8@ZIF-8 group compared with the LPS group. On the contrary, CD206 protein expression up-regulated under the stimulation of C_10_-KR8@ZIF-8. Although C_10_-KR8 promoted the M1 macrophages to a more anti-inflammatory phenotype, the C_10_-KR8@ZIF-8 nanoparticles modulated the LPS-damaged BMDMs to a state nearly identical to that of the control group (Fig. 4D to F). These findings indicated that C_10_-KR8@ZIF-8 nanoparticles can alleviate inflammatory conditions better than the C_10_-KR8 peptide. IF imaging was also conducted to validate this hypothesis. Various macrophage phenotype markers were presented in Fig. 4G and H. The LPS group exhibited CD86-positive macrophages (M1 marker), while the minimal number of CD206-positive cells was observed. However, in the C_10_-KR8@ZIF-8 groups, a contrasting pattern in which a greater proportion of M1 macrophages transitioned to the M2 phenotype emerged. The crucial involvement of C_10_-KR8@ZIF-8 in controlling macrophage polarization has been proven.

### C_10_-KR8@ZIF-8 nanoparticles promote the osteogenesis of BMSCs under inflammatory conditions

**Fig. 5.**
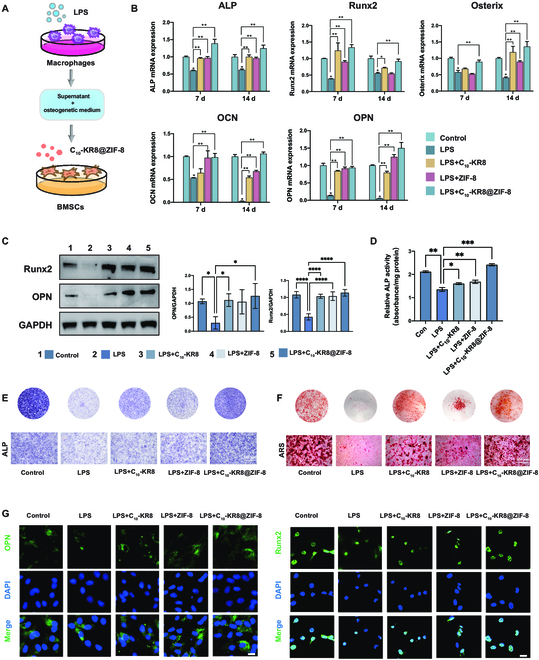
C_10_-KR8@ZIF-8 nanoparticles promote the osteogenic differentiation of BMSCs under inflammatory conditions. (A) An illustration in the form of a diagram. (B) Relative mRNA expression of the osteogenic genes in BMSCs stimulated with nanoparticles for 7 and 14 d. (C) Western blot analysis of Runx2 and OPN expression in BMSCs on day 14. (D) ALP activity of BMSCs stimulated with different nanoparticles. (E) ALP staining of BMSCs cultured with different nanoparticles for 7 d. Scale bar = 100 μm. (F) ARS staining of BMSCs cultured with different nanoparticles for 14 d. Scale bar = 100 μm. (G) Representative IF images of OPN+ and Runx2+ cells among the BMSCs. Scale bar = 50 μm. The data are presented as the mean ± SD (*n* = 3). ns, not significant; *P < 0.05, **P < 0.01.

To investigate the ability of the composite nanoparticles promoting bone formation under inflammatory conditions in vitro, we cultured BMSCs in CM from LPS-stimulated BMDMs (Fig. 5A). Our results demonstrated notable inhibition of osteogenesis-related mRNA and protein expression in the LPS group compared to the control group, consistent with prior findings (Fig. 5B and C).

qRT-PCR was used to further investigate the expression levels of osteogenesis-related genes in BMSCs cultured for 7 and 14 d. As shown in Fig. 5B, the mRNA levels of ALP, Runx2, OPN, OCN and Osterix were significantly up-regulated in both the C_10_-KR8 and ZIF-8 groups compared to those in the LPS group. Notably, the C_10_-KR8@ZIF-8 combination effectively reversed the inflammation-induced suppression of bone formation, nearly restoring the expression levels of these genes to those observed in BMSCs cultured in normal medium (Fig. 5B). Moreover, the Western blot results revealed the remarkable ability of C_10_-KR8@ZIF-8 to promote the osteogenic differentiation of BMSCs in inflammatory microenvironments. This was evident by the improved protein expression levels of Runx2 and OPN, supporting the findings from the qRT-PCR analyses (Fig. 5C).

To examine the early indicators of osteogenesis, ALP staining and ALP activity assays were performed, revealing an increasing trend in the expression of the nanocomplexes. Among the groups, the C_10_-KR8@ZIF-8 group exhibited the strongest staining intensity and ALP activity (Fig. 5D and E). To serve as a late marker of calcium deposition, ARS staining solution was used. Consequently, the ARS staining images displayed a trend similar to that of the ALP staining results. C_10_-KR8@ZIF-8 exhibited a greater capacity for the formation of mineralized calcium nodules than did C_10_-KR8 or ZIF-8 (Fig. 5F).

The ability of the C_10_-KR8@ZIF-8 group to enhance osteogenic differentiation was also confirmed through IF staining. Green and blue fluorescence markers were used to label the target proteins and nuclei, respectively. IF staining revealed higher expression levels of OPN and Runx2 in BMSCs from the C_10_-KR8, ZIF-8, and C_10_-KR8@ZIF-8 groups than in those from the LPS group. Notably, the C_10_-KR8@ZIF-8 group exhibited the strongest OPN and Runx2 fluorescence (Fig. 5G). These findings highlight the significant role of C_10_-KR8 in promoting BMSC osteogenesis, while the inclusion of ZIF-8 had synergistic effects on bone formation.

### C_10_-KR8@ZIF-8 interacts with Htra1 to promote osteogenesis under inflammatory conditions

**Fig. 6.**
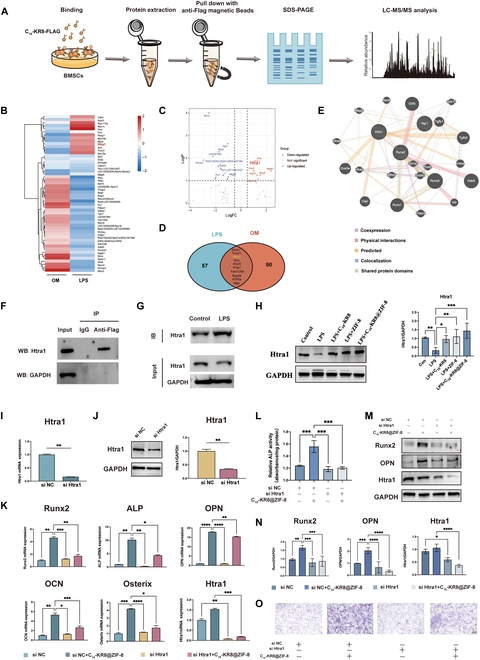
C_10_-KR8@ZIF-8 interacts with Htra1 to promote osteogenesis under inflammatory conditions. (A) Schematic diagram of the Co-IP/LC-MS/MS assay. (B) Heatmap of differentially expressed proteins pulled down by FLAG-labeled C_10_-KR8 in OM- and LPS-CM identified by MS. (C) Volcano plot of differentially expressed proteins pulled down by FLAG-labeled C_10_-KR8 in OM- and LPS-CM identified by MS. (D) Venn diagram of expressed proteins pulled down by FLAG-labeled C_10_-KR8 in OM- and LPS-CM identified by MS. (E) PPI network analysis. (F and G) Western blot analysis of Htra1 pulled down by FLAG-labeled C_10_-KR8 in OM- and LPS-CM. (H) Western blot and quantitation of the protein expression levels of Htra1 in BMSCs on day 7. (I and J) Expression levels of Htra1 mRNA and protein in BMSCs infected with si-NC or si-Htra1. (K) Expression of osteogenic genes in BMSCs infected with si-NC or si-Htra1transfection in LPS-CM. (L) Semiquantitative ALP activity of BMSCs infected with si-NC or si-Htra1 in LPS-CM. (M and N) Western blot analysis and quantification of Runx2, OPN, and Htra1 protein expressions infected with si-NC or si-Htra1 in LPS-CM. (O) ALP staining images of BMSCs infected with si-NC or si-Htra1in LPS-CM. The data are presented as the mean ± SD (*n* = 3). ns, not significant; *P < 0.05, **P < 0.01, and ***P < 0.001.

As a peptide, C_10_-KR8 may rely on specific binding to the cell membrane or intracellular targets to influence downstream signaling pathways and exert its effects. Previous studies have confirmed the bone-promoting effect of C_10_-KR8 within the inflammatory environment in vitro, which has significant implications for periodontal bone repair in periodontitis treatment. To determine the underlying mechanisms through which C_10_-KR8 enhances osteogenesis, we conducted a FLAG-labeled peptide pulldown/LC-MS/MS assay and proteomic analysis. These techniques aimed to identify target protein(s) that potentially interact with C_10_-KR8 (Fig. 6A).

We developed a Flag-labeled C_10_-KR8 strain (C10-KRIWQRIK-DYKDDDDK) to interact with BMSCs for 4 h in osteogenic medium (OM) or LPS-induced medium, and anti-Flag magnetic beads were used to isolate proteins bound to C_10_-KR8. Co-IP and LC-MS/MS experiments were subsequently conducted to identify the proteins that interact with C_10_-KR8 during BMSC osteogenesis under both inflammatory and noninflammatory conditions. We successfully identified 68 proteins that potentially interact with C_10_-KR8 during MSC osteogenesis and 76 proteins involved in inflammation. Figure 6B and C shows the heatmap and volcano plot, which visually represent the distribution and statistical significance of the differentially expressed proteins (DEPs) between the 2 groups. A Venn diagram of the results revealed the intersection of binding proteins between the 2 groups (Fig. 6D). Interestingly, Htra1 was found to be part of the Co-IP complex. Through PPI network analysis, we observed that the Htra1 protein may interact with several osteogenesis-related proteins, including Runx2, TGF-β, and Col1α (Fig. 6E).

We performed reciprocal Co-IP/Western blot assays to further validate the interaction between endogenous C_10_-KR8 and Htra1 in BMSCs. The immunoblotting results confirmed the specific interaction between C_10_-KR8 and Htra1 (Fig. 6F). Additionally, we observed an increase in the binding mass between C_10_-KR8 and Htra1 under LPS-stimulated inflammatory conditions compared to that under normal conditions (Fig. 6G). Western blot analysis revealed that Htra1 protein expression was significantly reduced in the inflammatory environment, but C_10_-KR8 improved Htra1 expression. Interestingly, ZIF-8 also promoted Htra1 protein expression, with the highest expression observed in the C_10_-KR8@ZIF-8 group (Fig. 6H). These analyses suggested that, in addition to the direct binding effect between C_10_-KR8 and Htra1, ZIF-8 may enhance Htra1 expression through other pathways. Therefore, C_10_-KR8@ZIF-8 may play dual roles in promoting bone formation, both directly and indirectly through Htra1.

To examine the involvement of Htra1 in facilitating the bone-forming function of C_10_-KR8@ZIF-8 in BMSCs, we used siRNAs to suppress the expression of Htra1. After verifying the ability of the siRNAs to inhibit both the RNA and protein levels of Htra1 (Fig. 6I and J), we investigated how the suppression of Htra1 affects the osteogenic differentiation of BMSCs in the presence of ZIFs. As shown in Fig. 6K and L, C_10_-KR8@ZIF-8 increased the expression of osteogenesis-related markers (ALP, Runx2, OCN, OPN, and Osterix) at the mRNA level and the ALP activity. However, these effects were significantly reduced in the si Htra1 group, suggesting that interference with Htra1 gene expression significantly impeded the C_10_-KR8@ZIF-8-induced osteoblastic differentiation of BMSCs. In line with these results, Western blot analysis showed that Htra1 knockdown decreased the protein levels of Runx2 and OPN (Fig. 6M and N), and the results of ALP staining showed that the ALP expression in BMSCs was consistent with the semiquantitative results of ALP activity (Fig. 6O). These results established Htra1 as a functional receptor for C_10_-KR8@ZIF-8 in BMSCs.

To further examine the involvement of Htra1 in osteogenic differentiation, BMSCs were subjected to Htra1 overexpression through short hairpin RNA. The overexpression of Htra1 was confirmed through Western blot analysis and qRT-PCR (Fig. S6A and B). Notably, the overexpression of Htra1 resulted in increased ALP activity and mRNA and protein expression of downstream osteogenesis-related genes, as illustrated in Fig. S6C to G. These findings strongly suggested that Htra1 functions as a crucial mediator of the response to C_10_-KR8@ZIF-8, as its expression was up-regulated by C_10_-KR8@ZIF-8, and Htra1 played a significant role in promoting the downstream osteogenic differentiation of BMSCs.

### The pathway by which C_10_-KR8@ZIF-8 promotes of osteogenesis under inflammatory conditions

**Fig. 7.**
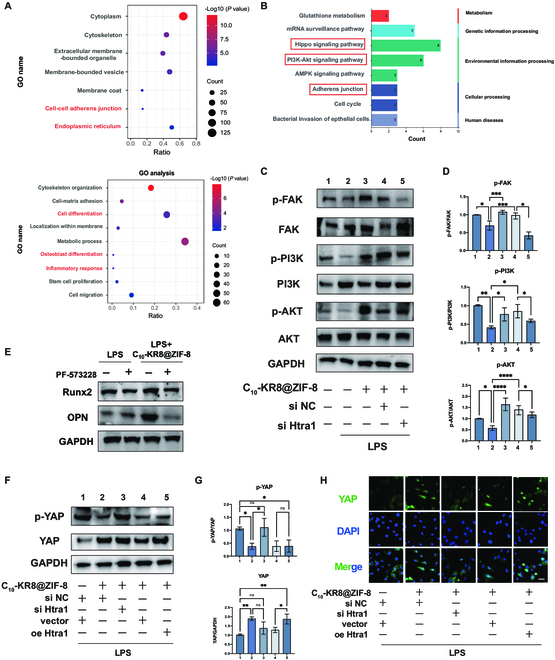
The pathway by which C_10_-KR8@ZIF-8 promotes osteogenesis under inflammatory conditions. (A) GO analysis of the enrichment of proteins differentially expressed between BMSCs with or without Htra1 knockdown. (B) KEGG classification diagram of proteins that are differentially expressed in BMSCs with or without Htra1 knockdown. (C and D) Western blot analysis and quantitation of the protein expression levels of p-FAK, FAK, p-PI3K, PI3K, p-AKT, and AKT in BMSCs. (E) Western blot analysis of the protein levels of Runx2 and OPN in BMSCs. (F and G) Western blot analysis and quantitation of the protein expression levels of p-YAP and YAP. (H) Representative IF images of YAP nuclear localization in BMSCs. Scale bar = 50 μm. The data are presented as the mean ± SD (*n* = 3). ns, not significant; *P < 0.05, **P < 0.01, ***P < 0.001, and ****P < 0.0001.

To explore the mechanism underlying C_10_-KR8@ZIF-8-mediated osteogenic differentiation through Htra1, we performed proteomic analysis of BMSCs transfected with si-NC or si-Htra1 using high-resolution LC-MS/MS technology. This analysis aimed to identify differences in the proteomic signatures of BMSCs cultured under inflammatory conditions and treated with C_10_-KR8@ZIF-8 with or without Htra1 knockdown. GO enrichment analysis and GO annotation revealed that several differentially expressed proteins were enriched and involved in several critical functions of BMSCs during the process of osteogenic differentiation, including cell proliferation, cell migration, and osteogenesis (Fig. 7A). To obtain functional annotation information for the differentially expressed proteins, KEGG enrichment analysis was conducted. According to Fig. 7B, the KEGG enrichment analysis indicated a strong association between the differentially expressed proteins in C_10_-KR8@ZIF-8 and the Hippo and PI3K/AKT signaling pathways, which are recognized to play a role in osteogenic differentiation.

KEGG enrichment analysis also revealed that the adherens junction pathway was involved in the osteogenic process stimulated by C_10_-KR8@ZIF-8. Western blot analysis indicated that C_10_-KR8@ZIF-8 treatment augmented the expression of phosphorylated FAK protein. Moreover, knockdown of Htra1 inhibited the phosphorylation of FAK (Fig. 7C and D). Moreover, the chemical inhibitor FAK PF-573228 was used to confirm the functional significance of FAK activation in C_10_-KR8@ZIF-8-induced osteogenic differentiation. Subsequent protein analysis demonstrated substantial down-regulation of Runx2 and OPN expression in inflammatory environments, regardless of the presence or absence of C_10_-KR8@ZIF-8 treatment (Fig. 7E).

Given that FAK is closely connected with YAP, which is also a pivotal molecule in the Hippo pathway, we investigated whether C_10_-KR8@ZIF-8 can influence Htra1-mediated YAP activation. Western blot and IF analyses demonstrated that in an inflammatory state, C_10_-KR8@ZIF-8 inhibited YAP activation and induced YAP translocation into the nucleus. To investigate the potential role of Htra1 in mediating the association between C_10_-KR8@ZIF-8 stimulation and YAP activation, which promotes BMSC osteogenesis, we down-regulated Htra1 expression in BMSCs. Htra1 depletion resulted in reduced YAP phosphorylation and decreased YAP expression in cells. Conversely, Htra1 overexpression led to increased YAP nuclear localization. These findings demonstrated that C_10_-KR8@ZIF-8 modulates osteogenesis by activating the Htra1/FAK/YAP signaling pathway (Fig. 7F to H).

Moreover, through Western blot analysis, we observed significant increases in the levels of the phosphorylated AKT and PI3K proteins in BMSCs following treatment with C_10_-KR8@ZIF-8. Notably, the increase in p-PI3K and p-AKT induced by C_10_-KR8@ZIF-8 decreased after Htra1 was knocked down (Fig. 7D). Taken together, these findings suggested that the combined action of C_10_-KR8 and ZIF-8, known as C_10_-KR8@ZIF-8, partially regulated osteogenic differentiation by activating the PI3K/AKT signaling pathway.

### Injectable GelMA hydrogels loaded with C_10_-KR8@ZIF-8 reverse alveolar bone loss in a rat model of ligature-induced periodontitis

**Fig. 8.**
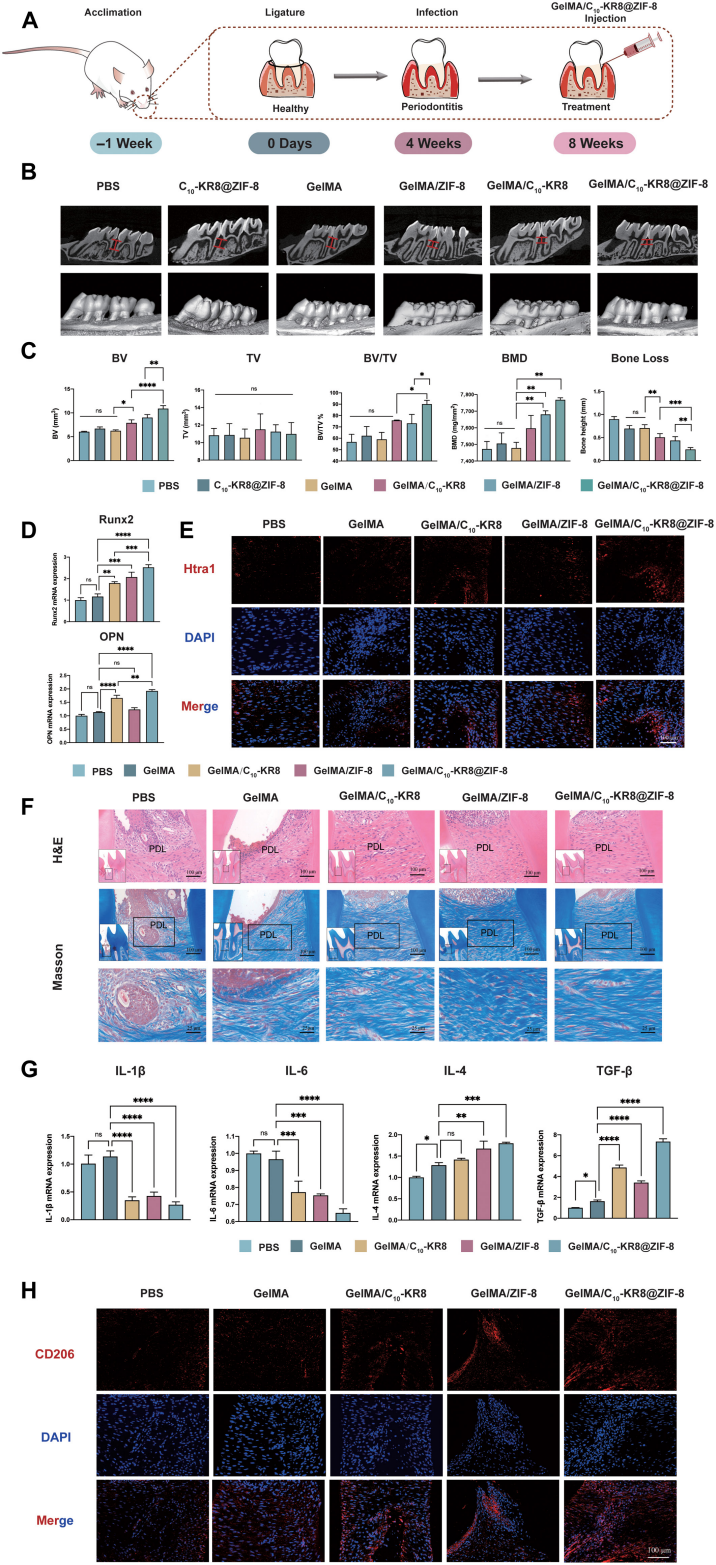
Injectable GelMA hydrogels loaded with C_10_-KR8@ZIF-8 reverse alveolar bone loss in a ligature-induced periodontitis rat model. (A) A schematic illustration of the ligature-induced periodontitis rat model. (B) 3D reconstructed images and bucco-palatal section images of maxillary molars analyzed by micro-CT. Red boxes represent the distance between ABC and CEJ (scale bar = 1 mm). (C) Quantitative statistics obtained for different parameters related to the alveolar bone. (D) Gene expression levels of the Runx2 and OPN in the bone surrounding the upper first molars determined via qRT-PCR. (E) IF staining of Htra1 in the periodontal tissue of rats. (F) H&E and Masson staining images of the periodontal tissue. Scale bar = 100 μm. (G) Expression of the inflammation-related genes in the bone around the upper first molars. (H) IF staining of CD206 in the periodontal tissue of rats. The data are presented as the mean ± SD (*n* = 5). ns, not significant; *P < 0.05, **P < 0.01, ***P < 0.001, and ****P < 0.0001.

Given the significant effects of C_10_-KR8@ZIF-8 on immune regulation and osteogenic differentiation in BMSCs, we undertook additional studies to further validate the interventionist effects of C_10_-KR8@ZIF-8 on the regeneration of inflamed periodontal tissue in vivo (Fig. 8A).

Overall, the combination of ligation effectively triggered periodontitis in Sprague Dawley rats, leading to the deterioration of bone structure and decreased height of the bone surrounding the teeth (in the PBS group). In these rats with periodontitis, there was severe resorption of the alveolar bone, significant recession of the gingiva below the enamel-cementum boundary, and clear exposure of the roots, indicating severe periodontal lesions (Fig. 8B). However, the directly injection of C_10_-KR8@ZIF-8 nanoparticles to periodontitis area did not show significant bone remodeling effect. The bone mass and bone height measurements of C_10_-KR8@ZIF-8 group showed no significance compared with the PBS group (Fig. 8B and C). This may be due to the fact that nanoparticles cannot stay in periodontal pockets for a long time, and the nanoparticles are metabolized during the feeding process of rats.

To overcome the challenge of securing peptides and nanoparticles in the periodontal pocket, GelMA hydrogels were employed as scaffolds for loading C_10_-KR8, ZIF-8, and C_10_-KR8@ZIF-8. These hydrogels were subsequently injured into gingival pockets located between the first and second molars of rats with ligature-induced periodontitis. The bone volume, tissue volume, bone volume/tissue volume, and bone loss were measured (Fig. 8B and C) via micro-CT analysis in addition to 3D reconstructed images. The administration of PBS and GelMA into the gingival pockets did not relieve periodontal bone loss, whereas the GelMA/C_10_-KR8 and GelMA/ZIF-8 groups exhibited alleviation of the symptoms associated with periodontal bone loss. The GelMA/C_10_-KR8@ZIF-8 group demonstrated the highest level of alveolar bone height restoration, with abundant bone filling observed in the root furcation regions. These findings illustrated the collaborative impact of C_10_-KR8 and ZIF-8 on enhancing the regeneration of alveolar bone in vivo.

To further explore the impact of C_10_-KR8@ZIF-8 on bone regeneration, we collected fresh bone tissue adjacent to the first molar for qRT-PCR analysis (Fig. 8D). The Runx2 and OPN mRNA expression levels in the GelMA/C_10_-KR8@ZIF-8 group exhibited a notable increase compared to those in the remaining groups, indicating the effective promotion of osteogenesis by C_10_-KR8, which was further enhanced by the presence of Zn ions. Therefore, these results suggest that C_10_-KR8@ZIF-8 exhibits excellent osteogenic properties in periodontitis. Furthermore, IF staining of Htra1 in periodontal tissue confirmed that the GelMA/C_10_-KR8@ZIF-8 scaffold prominently promoted the expression of Htra1, which was consistent with the in vitro results (Fig. 8E).

H&E and Masson staining showed that the PBS and GelMA groups experienced fibrous degeneration and degradation due to inflammation, leading to a disarrayed and scarce distribution of fibers within the periodontal tissue, as well as a loss of alveolar bone. Conversely, the GelMA/C_10_-KR8-, GelMA/ZIF-8-, and GelMA/C_10_-KR8@ZIF-8-treated groups exhibited fewer inflammatory cells, more substantial layers of epithelial cells, denser and more well-arranged elastic and collagen fibers, and more alveolar bone (Fig. 8F). Notably, the GelMA/C_10_-KR8@ZIF-8 group exhibited PDL fibers in a highly consistent orientation, reestablishing the physiological slope and indicating the recovery of periodontal function.

In view of the positive results for new bone formation with GelMA/C_10_-KR8@ZIF-8 nanoparticles, our objective was to examine the immune mechanism responsible for in vivo periodontal bone deterioration. In this study, we collected fresh bone tissue adjacent to the first molar. The increased mRNA expression of IL-1β and IL-6 in the PBS group suggested the activation of M1 macrophages and an abundance of inflammatory cytokines in periodontitis. Treatment with GelMA/C_10_-KR8 led to a decrease in the relative mRNA expression levels of IL-6 and IL-1β. Additionally, the addition of ZIF-8 nanoparticles enhanced this impact, resulting in the lowest levels being observed in the GelMA/C_10_-KR8@ZIF-8 group. In contrast, the mRNA expression of IL-4 and TGF-β was the highest in the GelMA/C_10_-KR8@ZIF-8 group (Fig. 8G).

Fluorescence staining of CD206 was conducted to determine M2 macrophage polarization. The GelMA/C_10_-KR8@ZIF-8 group exhibited the highest CD206 protein expression, suggesting a notable inclination toward M2 polarization (Fig. 8H). In the periodontal region, C_10_-KR8@ZIF-8 showed potential therapeutic benefits by inhibiting inflammation and facilitating bone regeneration, thus demonstrating its efficacy in treating plaque-induced periodontitis.

## Discussion

Periodontitis is a chronic inflammatory disease initiated by gram-negative or gram-positive bacteria and accompanied by immune environment disorders and excessive production of reactive oxygen species, resulting in alveolar bone resorption and tooth loss [1]. LL-37 as an AMP has antibacterial properties, immunomodulatory functions, and bone healing functions under physiological conditions such as periodontitis [30]. However, the use of the LL-37 peptide in therapeutics is impeded by its low serum stability and relatively high manufacturing costs [16]. Therefore, this study finds the smallest active unit of LL-37, KR-8, and conjugated it with C10 fatty acid to enhance peptide–membrane interactions and stabilities.

In periodontitis treatment, how to regulate immune disorders and promote bone formation is still a problem that needs to be solved. Macrophages play vital roles in modulating the immune system and maintaining periodontal environment homeostasis [31]. Given the functional versatility of macrophages, an imbalance in M1/M2 phenotype macrophages may aggravate periodontal tissue damage [32]. In our study, we observed that C_10_-KR8 exhibited excellent anti-inflammatory effects. Treatment of LPS-activated macrophages with C_10_-KR8 led to a reduction in the initial abundance of M1 markers, such as iNOS and CD86, while increasing the expression of the relatively low M2 phenotype markers Arg-1 and CD206. These findings suggest that C_10_-KR8 can induce the polarization of M1-type macrophages toward the M2 phenotype to achieve homeostasis of the immune environment, which is also important for the regulation of osteoimmunology and enhancement of bone repair in periodontitis.

The interaction between macrophages and MSCs plays a crucial role in bone repair during periodontitis [33]. Several studies have indicated that proinflammatory factors released by M1 macrophages, such as IL-6 and IL-1β, can inhibit osteogenic differentiation. In contrast, M2 macrophages can secrete anti-inflammatory factors and growth factors, such as IL-4 and IL-10, to support MSC-mediated bone regeneration, which is closely associated with tissue repair and bone formation [29,34]. In our research, we developed a coculture system involving macrophages and BMSCs, which enabled the addition of CM with soluble substances from macrophages to indirectly induce cellular interactions resembling the inflammatory environment in periodontitis. The osteogenic differentiation of BMSCs was significantly inhibited by the inflammatory microenvironment induced by LPS, possibly because of the presence of proinflammatory substances in the CM discharged by M1 phenotype macrophages. Our findings demonstrated that the C_10_-KR8 peptide enhanced the osteogenic differentiation of BMSCs within a short timeframe by regulating the inflammatory microenvironment. Furthermore, in the inflammatory microenvironment induced by LPS-activated macrophages, C_10_-KR8 can directly activate BMSCs to promoted long-term osteogenesis. Overall, our study demonstrates the effective promotion of osteogenic differentiation and mineralization by C_10_-KR8 through influencing the communication between macrophages and BMSCs.

Despite its excellent biological regulatory ability, C_10_-KR8 exhibits limited stability and a short half-life due to enzymatic degradation and rapid kidney filtration [14]. To overcome these limitations and achieve the desired therapeutic effect, one promising solution is to incorporate the peptide into a suitable carrier to enhance its stability and mediate desirable drug release to avoid repeated doses. ZIF-8, a member of the MOF subclass, is an excellent candidate for this purpose. ZIF-8 is biocompatible, has customizable pore openings, and can be successfully applied in drug delivery [35]. It also has the ability to protect biomacromolecules [19]. Moreover, ZIF-8 is pH sensitive, allowing easier release and degradation in acidic environments, such as those found in infected tissues [36]. In this study, C_10_-KR8@ZIF-8 nanoparticles were developed with classical regular dodecahedron structures and sharp edges, and long-term stable release of C_10_-KR8 was achieved. Moreover, we observed that, compared with C_10_-KR8 alone, C_10_-KR8@ZIF-8 had stronger anti-inflammatory activity, more pronounced macrophage reprogramming, and greater osteogenic activity in BMSCs under inflammatory conditions. These enhanced activities may be attributed to the sustained release of Zn^2+^ from ZIF-8, which has been reported to regulate macrophage polarization and promote osteogenic differentiation in MSCs [23,37]. In the rat model of periodontitis in this study, the treatment of periodontitis by injecting C_10_-KR8@ZIF-8 nanoparticles alone did not achieve good therapeutic effect. This may be because nanoparticles are easy to be metabolized and difficult to release at fixed sites in vivo. To stabilize C_10_-KR8@ZIF-8 nanoparticles in the periodontal pocket for a long time, a GelMA hydrogel was used as a scaffold to fill irregular periodontal defects in vivo. The findings provide further evidence of the immunomodulatory and osteogenic potential of C_10_-KR8@ZIF-8 nanoparticles in vivo, as they reduce the frequency of clinical administration at the same time.

To investigate the mechanism through which C_10_-KR8 and C_10_-KR8@ZIF-8 regulate bone formation in an inflammatory environment, Co-IP/LC-MS/MS analysis was conducted. In this study, the C_10_-KR8 peptide was conjugated with FLAG tags, which were genetically engineered to form a fusion protein with a specific amino acid sequence (DYKDDDDK) and attached to the target protein [38]. We utilized anti-Flag magnetic beads to isolate proteins that are bound to C_10_-KR8-FLAG. Mass spectrum (MS) analysis revealed that C_10_-KR8 may bind to the Htra1 protein. Htra1 is a serine protease involved in a wide range of cellular functions and is implicated in various disease processes, including osteogenesis and bone differentiation [39]. Multiple studies have suggested that Htra1 can influence the expression of genes and signaling pathways associated with osteogenic differentiation and affect the delicate balance between bone formation and resorption, which is crucial for maintaining bone health [40,41]. In our study, Co-IP/Western blot analysis revealed an interaction between C_10_-KR8 and the Htra1 protein, and both C_10_-KR8 and ZIF-8 enhanced Htra1 expression in LPS-induced conditioned media. Furthermore, the knockdown and overexpression of Htra1 significantly affected the osteogenic differentiation ability of C_10_-KR8@ZIF-8 cells. Taken together, these findings indicate that C_10_-KR8@ZIF-8 enhances osteogenic differentiation by binding with Htra1 and inducing its expression.

KEGG enrichment analysis of differentially expressed proteins in C_10_-KR8@ZIF-8-treated BMSCs with or without Htra1 knockdown revealed that C_10_-KR8@ZIF-8 potentially influences the adherens junction pathway through Htra1. Inflammation has been shown to influence cell adhesion [42], and our study demonstrated that cell morphology shrinks when cells are cultured in an inflammatory environment. However, in the C_10_-KR8@ZIF-8 group, the BMSCs exhibited a spread cell morphology and reversal of the inflammation-induced adverse effects on cell adhesion. These findings provide evidence of the remarkable adhesive and morphological extension capabilities of BMSCs, which can be attributed to the presence of C_10_-KR8@ZIF-8, even in the presence of an inflammatory microenvironment. FAK plays a pivotal role in the transduction of signals mediated by integrins, regulating the migration, adhesion, and differentiation of MSCs [43–45]. In our study, C_10_-KR8@ZIF-8 significantly enhanced FAK phosphorylation, and Htra1 knockdown reversed this effect.

FAK is closely connected with YAP [46], which is also a pivotal molecule in the Hippo pathway, and studies have indicated a significant relationship between the activity level of YAP and the determination of bone cell fate [47]. Our study finds that C_10_-KR8@ZIF-8 may enhance Htra1 expression, thereby promoting YAP activation and nuclear localization. Consequently, this may activate acetylation of the osteogenic marker Runx2 promoter, ultimately enhancing the osteogenic differentiation of BMSCs [48]. Multiple studies have consistently demonstrated the vital importance of the PI3K/AKT signaling pathway in the process of osteogenic differentiation [49,50]. In the present study, KEGG pathway enrichment showed that the PI3K/AKT pathway was suppressed after Htra1 was knocked down. Western blot analysis also revealed that C_10_-KR8@ZIF-8 activated the PI3K/AKT pathway, and Htra1 knockdown reversed this effect.

Collectively, our results indicated that the osteogenic function of the C_10_-KR8 peptide is a result of its direct binding to Htra1, while ZIF-8 indirectly increases Htra1 expression, thereby influencing FAK activation and YAP nuclear localization. Knocking down Htra1 also affects the PI3K/AKT pathway, indicating its potential involvement in C_10_-KR8@ZIF-8-mediated regulation of osteogenesis under inflammatory conditions.

However, before C_10_-KR8@ZIF-8 nanoparticles can be utilized in clinical settings, certain practical concerns need to be addressed, including the safe manufacturing and quality control of nanoparticles. Moreover, we believe that further optimization of biomaterials is necessary to achieve effective therapeutic outcomes, as is the development of scalable mass-production solutions. There is still a clear demand for advancements in optimized technologies, which will facilitate the clinical application of C_10_-KR8@ZIF-8 for the treatment of periodontitis.

### Conclusion

Treatment of periodontitis is a long-standing and urgent clinical problem in which bone regeneration is achieved by modulating the immune microenvironment and improving osteogenic differentiation. In this study, for the first time, we confirmed that the fatty acid-modified polypeptide C_10_-KR8 has excellent ability to modulate the communication between macrophages and BMSCs, resulting in improved osteogenesis. Additionally, we developed C_10_-KR8@ZIF-8 nanoparticles that specifically target the pathological characteristics of the periodontitis microenvironment. Under inflammatory conditions, these nanoparticles exhibit sustained drug release and release Zn ions from ZIF-8. This action collaboratively targets inflammatory areas, stimulates immune reprogramming to facilitate macrophage repolarization, and restores osteogenesis in vitro and in vivo. To investigate the binding target of C_10_-KR8, we conducted a FLAG-labeled peptide pulldown/MS assay and proteomic analysis, which revealed an interaction between C_10_-KR8 and Htra1. Furthermore, C_10_-KR8@ZIF-8 promoted BMSC differentiation and mineralization through the Htra1/FAK/YAP pathway. Additionally, the FAK/PI3K/AKT signaling pathway was partially activated, contributing to this process. In summary, this research not only clarified the effect of the C_10_-KR8 peptide on periodontal regeneration but also created a promising platform for delivering peptides, C_10_-KR8@ZIF-8, to enhance the therapeutic efficacy of periodontitis treatment. In the future, the increased incorporation of biomaterials such as hydrogels or electrospun fibrous mats is expected to lead to the customization of our delivery platform for periodontitis therapy in individual patients. This approach holds potential for use as a highly adaptable technology in clinical applications.

## Data Availability

The data are available from the corresponding author on reasonable request.
