# Supplementary material for: A Novel Lipopeptide–Functionalized Metal–Organic Framework for Periodontitis Therapy through the Htra1/FAK/YAP Pathway
Source: Biomater Res. 2024 Jul 29;28:0057. doi: 10.34133/bmr.0057 (PMC11283871; doi:10.34133/bmr.0057)
Supplement: Supplementary 1 — Figs. S1 to S6 Tables S1 to S4 [file bmr.0057.f1.docx]

**Supporting Information**

**A Novel Lipopeptide–Functionalized Metal–Organic Framework for Periodontitis Therapy through the Htra1/FAK/YAP Pathway**

1. **Materials and Methods**

**Alkaline phosphatase Staining and Activity**

Alkaline phosphatase (ALP) staining was also conducted to further examine the conditions of cellular osteogenic differentiation. BMSCs were incubated with various types of conditioned media for 7 days. Next, the cells in the 24-well plates were treated with 4% paraformaldehyde (Sigma‒Aldrich Co., USA) for 20 minutes and subsequently washed with PBS. Next, the cells were treated with an ALP kit (Beyotime) for 30 minutes and subsequently observed using an inverted light microscope. For quantitative analysis of ALP activity, the supernatants of the lysed cells were collected by centrifugation. Subsequently, the chromogenic reagent was added following the instructions provided with the ALP detection kit (Beyotime). The absorbance of each sample was detected at 405 nm, and the ALP activity in the samples was normalized to the total protein concentration.

**Alizarin red S Staining**

ARS staining was also used to evaluate osteogenic differentiation. Following a 14-day incubation with various types of CM, the cells were treated with 4% paraformaldehyde for fixation and subsequently washed with PBS. Then, the cells were stained with 1% ARS (Cyagen Biosciences, Inc., USA) solution for a suitable time in an incubator at 37°C. After incubation, the excess of ARS was removed by rinsing with distilled water.

**Enzyme-Linked Immunosorbent Assay**

Macrophages were cultured with different treatment, The supernatant sample was collected and subsequently centrifuged at a speed of 3,000 rpm for 15 minutes. The concentration of IL-1β, IL-6, IL-4, and IL-10 was detected using ELISA kits (Boster, China) in accordance with the instructions provided by the manufacturer.

**Flow Cytometry**

After being washed in PBS, the RAW264.7 cells and BMDMs were collected and fixed in 80% ethanol at a low temperature. Afterward, the cells were centrifuged at a low temperature and reconstituted in cold PBS. Next, the cells were cultured with CD86 (BioLegend, USA) and CD206 (BioLegend, USA) for 30 minutes at 4°C. The cells were subsequently detected using a flow cytometer (BD FACS Calibur). FlowJo software was used to analyze the data.

**IF Assay**

Cells were seeded on sterile glass coverslips. The specimens were fixed in 4% paraformaldehyde (PFA) for half an hour and then treated with 0.1% Triton X-100 for twenty minutes to allow permeabilization. Subsequently, the cells were blocked using a 1% BSA solution for 1 h. Furthermore, the specimens were incubated with the primary antibody (Table S2) at 4°C overnight. Next, the cells were incubated with a secondary antibody for 1 h at 37°C and then incubated with DAPI for 10 minutes at room temperature. CLSM was used to observe the samples.

**Drug release experiment**

To generate a standard curve, C10-KR8 was detected using a bicinchoninic acid (BCA) Protein Assay Kit (Beyotime, China) at concentrations of 0 μg/ml, 15.625 μg/ml, 31.25 μg/ml, 62.5 μg/ml, 125 μg/ml, 250 μg/ml, 500 μg/ml, 1000 μg/ml, and 2000 μg/ml. Subsequently, 5 mg of C10-KR8@ZIF-8 was submerged in PBS at pH 7.4 and pH 5.5 with a stirring speed of 100 rpm at 37°C for 1, 2, 3, 5, 7, 10, and 14 days. After being collected, all the solutions were evaluated using BCA analysis. At each time point, the presence of Zn ions was identified using inductively coupled plasma‒optical emission spectrometry (ICP‒OES).

**Micro-CT evaluation**

The maxillae of SD rats were dissected and subjected to micro-CT imaging system (Quantum GX, PerkinElmer, USA). 3D digital images were reconstructed and cross-sectional images in bucco-lingual directions were obtained with Slicer software.

The region of interest (ROI) was set was set at the bone area around and the the root bifurcation area of the maxillary first molars, excluding the teeth. Bone mass was assessed using CAn software, which calculated the ratios of bone volume to total volume (BV/TV) and bone mineral density (BMD) around the ligated molar. Bone loss was defined as the distance between the alveolar bone crest (ABC) to the cementoenamel junction (CEJ).

**Histological Analysis**

For histological analysis, the maxillae of experimental rats were fixed and decalcified with 10% EDTA for 6 weeks. using 10% EDTA solution for a duration of 6 weeks. Tissue sections with a thickness of approximately 5 μm were prepared after embedding the slices. The histological alterations in the periodontal tissue were evaluated using H&E (Solarbio, China) and Masson (Solarbio) staining.

1. **Results**


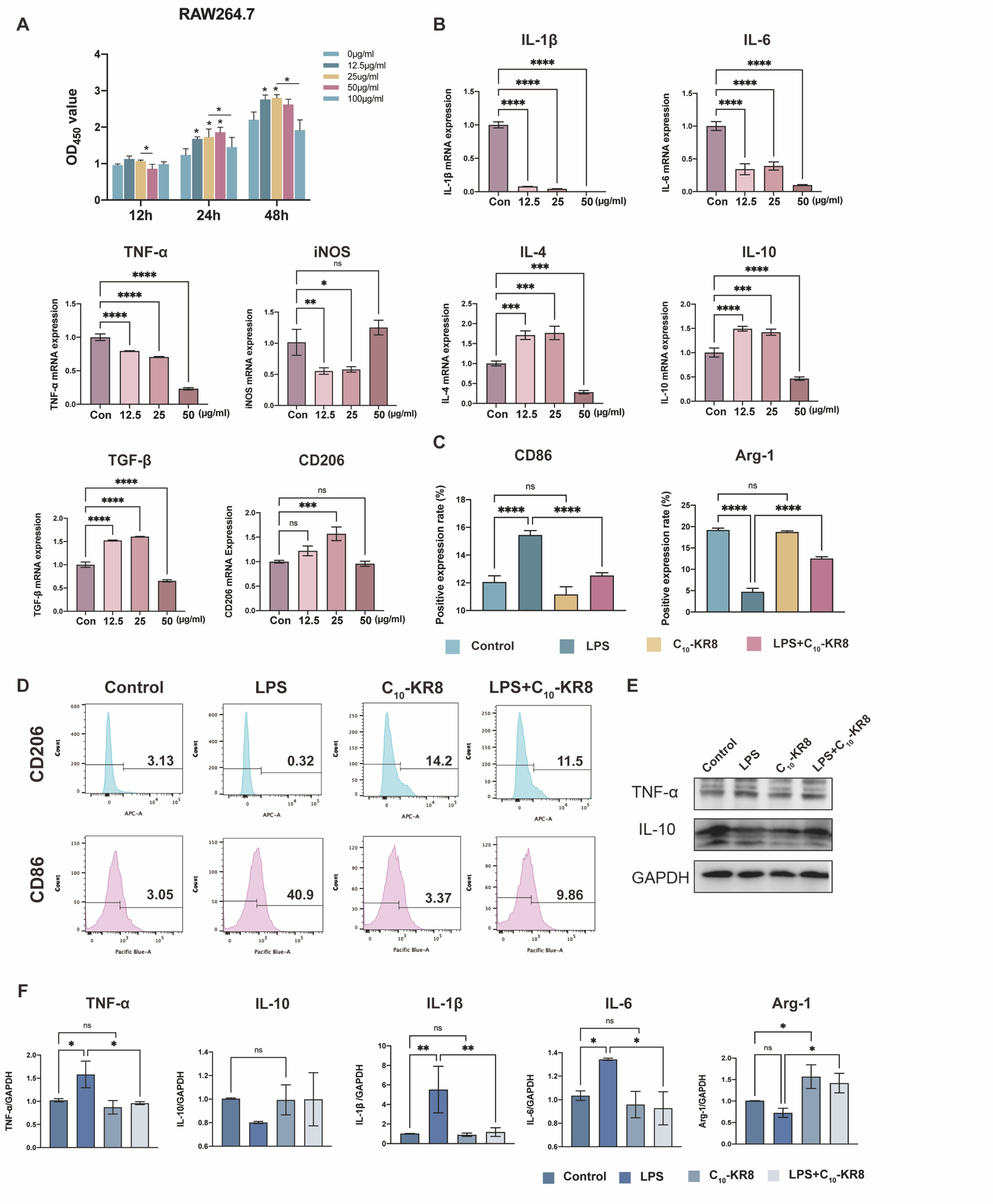


**Fig. S1.** The effects of C_10_-KR8 on immunomodulation. (A) Cell proliferation of RAW264.7 cells stimulated by gradient concentration of C_10_-KR8 evaluated by CCK-8 kit on 12h, 24h, and 48h. (B) Gene expressions of the pro-inflammatory cytokines (IL-1β, IL-6, TNF-α and iNOS) and the anti-inflammatory cytokines (IL-4, IL-10, CD206 and TGF-β) measured by qRT-PCR. (C) Semiquantitative statistical analyses of IF images of CD86+ and Arg-1+ cells. (D) Flow cytometry analysis of CD86 and CD206 expression of RAW264.7 cells. (E) Expression of macrophage polarization-related protein (TNF-α and IL-10) in RAW264.7 cells (Western blot). (F) Quantitative analysis (normalized to -GAPDH) of the relative band intensity (TNF-α, IL-10, IL-1β, IL-6, and Arg**-**1). The data are presented as the mean ± SD (n= 3). ns: no significance; **p* < 0.05, ** *p* < 0.01, *** *p* < 0.001 and **** *p* < 0.0001.


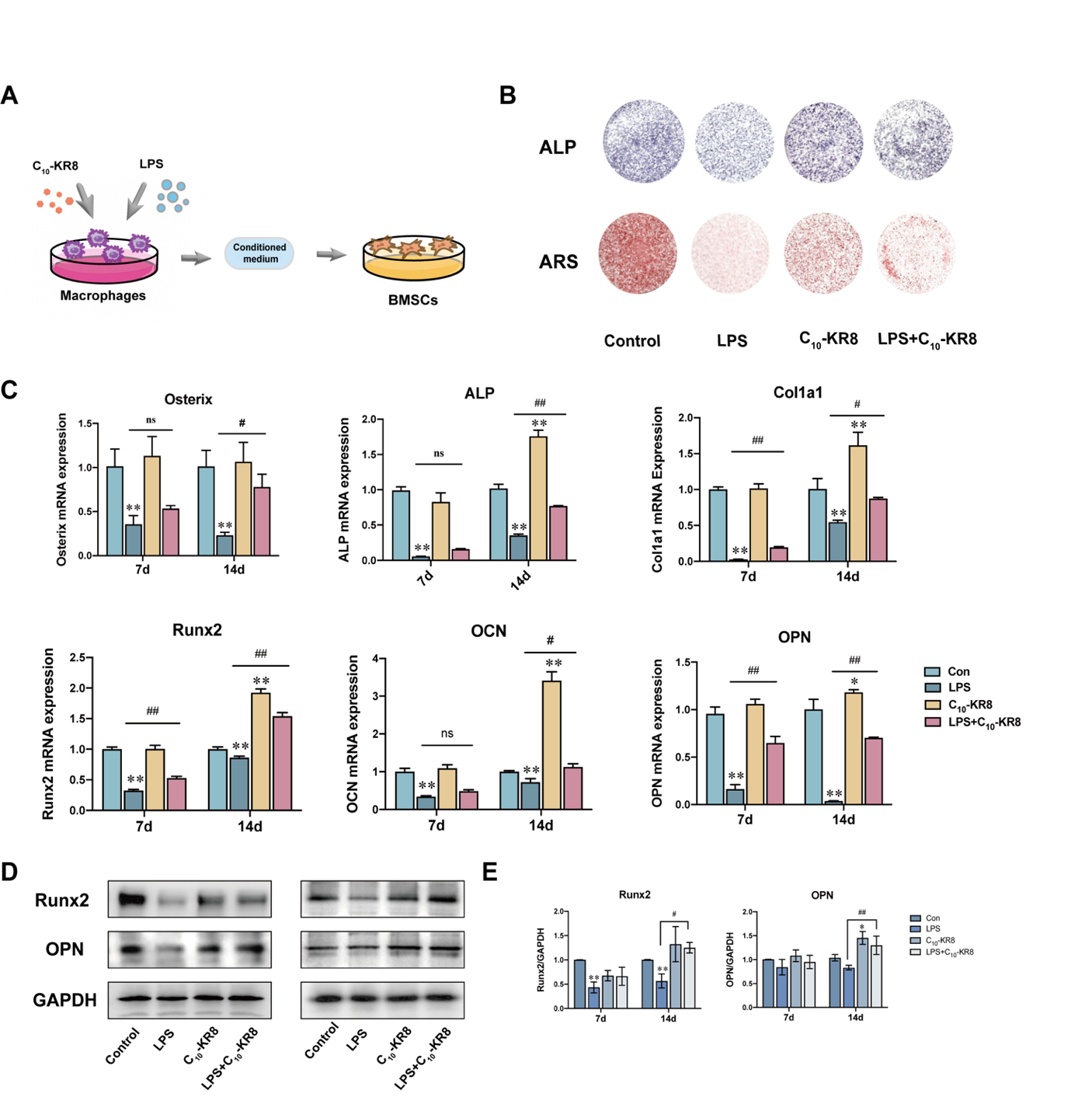


**Fig. S2.** C_10_-KR8 significantly enhanced the osteogenic differentiation through regulating osteoimmunology. (A) Schematic illustration. (B) ALP staining pictures of the BMSCs cultured in corresponding CM for 7 days, and ARS image analysis of the BMSCs cultured with corresponding CM for 14 days. (C) Relative mRNA expression of osteogenic genes Osterix, ALP, Col1α1, Runx2, OCN and OPN in BMSCs stimulated by different CM for 7and 14 days. (D) Western blot results of Runx-2 and OPN expression in BMSCs on day 7 and 14. (E) Quantitation of the protein expression levels of Runx2 and OPN. The data are presented as the mean ± SD (n= 3). ns: no significance; **p* < 0.05, ** *p* < 0.01 compared with the control group; ^#^*p* < 0.05, ^##^ *p* < 0.01 compared with the LPS group.


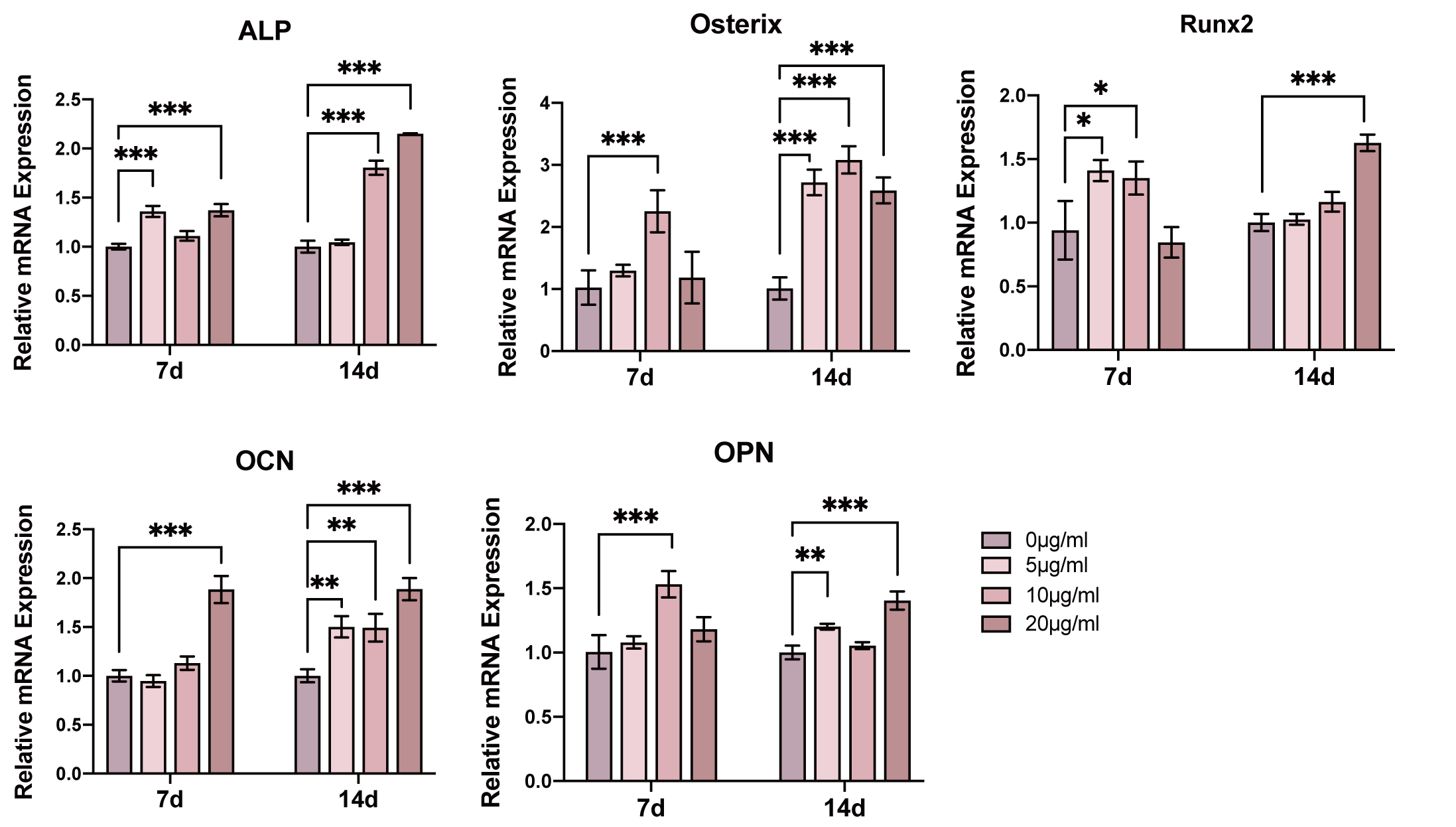


**Fig. S3.** Relative mRNA expressions of osteogenic gene Osterix, ALP, Runx2, OCN and OPN in BMSCs stimulated by gradient concentration of C_10_-KR8 stimulation for 7 and 14 days. The data are presented as the mean ± SD (n= 3). **p* < 0.05, ** *p* < 0.01, *** *p* < 0.001.


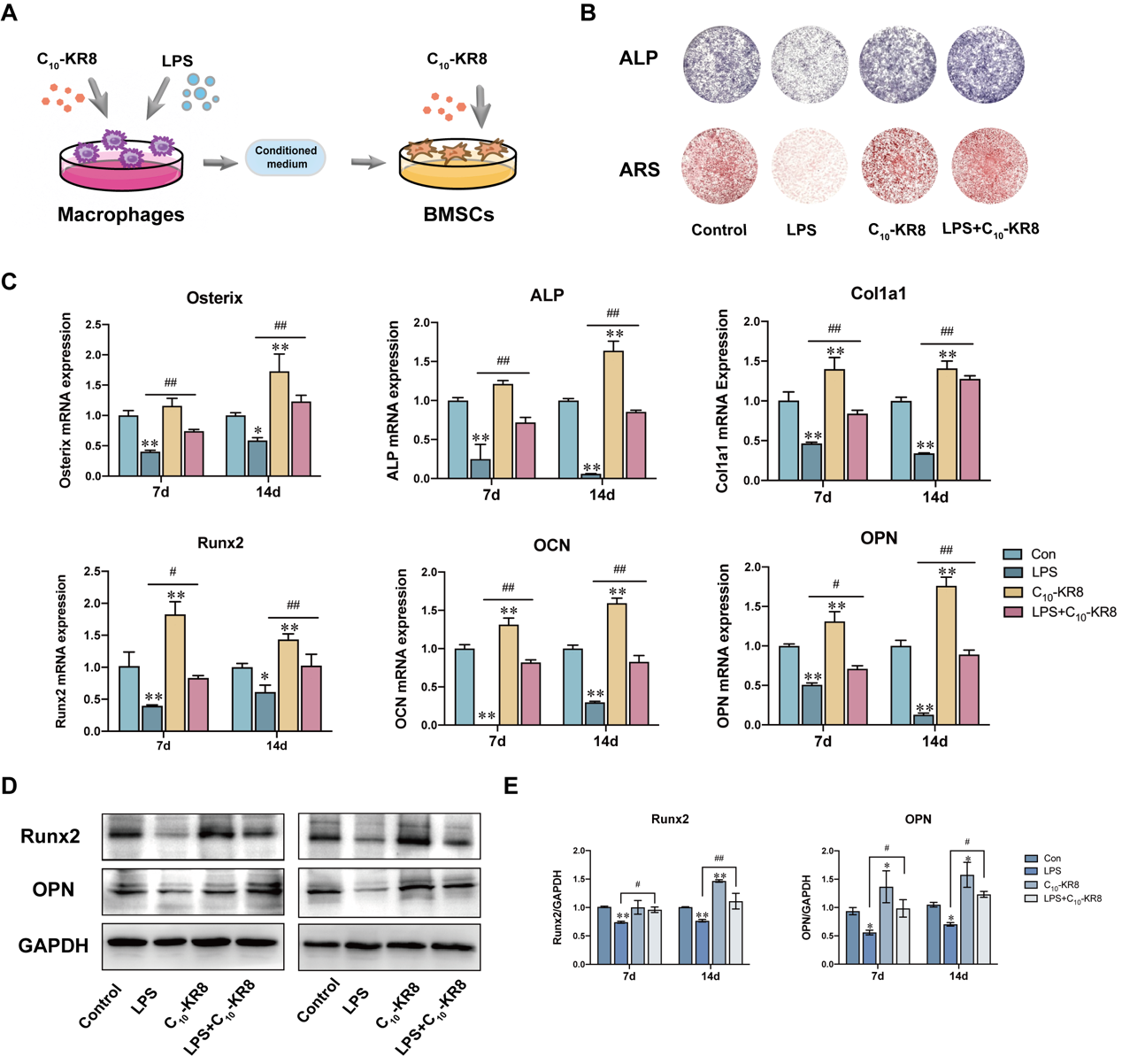


**Fig. S4.** C_10_-KR8 significantly enhanced the osteogenic differentiation through modulating both macrophage and BMSCs. (A) Schematic illustration. (B) ALP staining pictures of the BMSCs cultured in corresponding CM for 7 days, and ARS image analysis of the BMSCs cultured with corresponding CM for 14 days. (C) Relative mRNA expression of osteogenic genes Osterix, ALP, Col1α, Runx2, OCN and OPN in BMSCs stimulated by different CM for 7and 14 days. (D) Western blot results of Runx-2 and OPN expression in BMSCs on day 7 and 14. (E) Quantitation of the protein expression levels of Runx2 and OPN. The data are presented as the mean ± SD (n= 3). ns: no significance; **p* < 0.05, ** *p* < 0.01 compared with the control group; ^#^*p* < 0.05, ^##^ *p* < 0.01 compared with the LPS group.


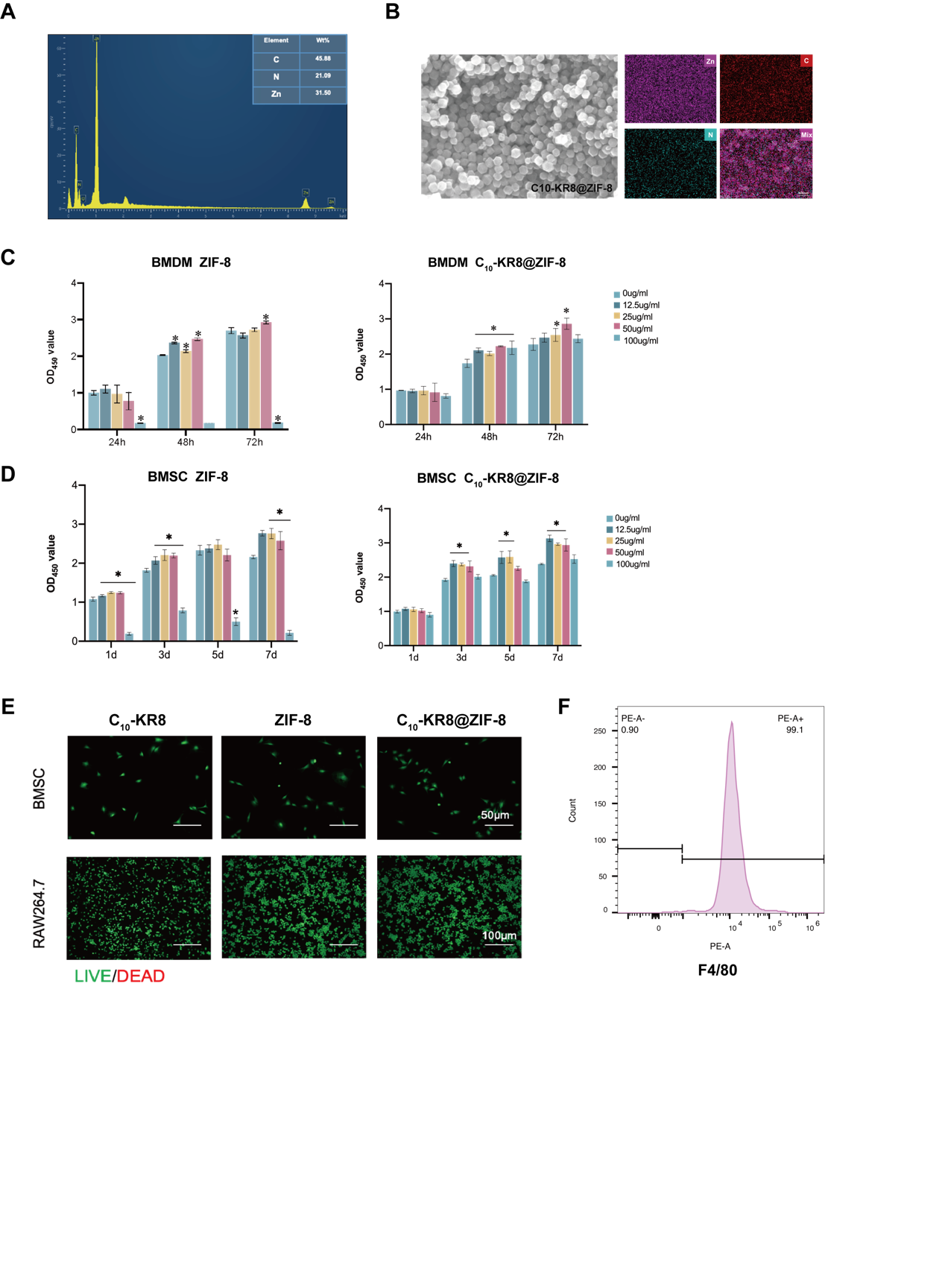


**Fig. S5.** Composition and characterization of MOFs. (A) EDS image of C_10_-KR8@ZIF-8 nanoparticles. (B) Elemental mapping images of the C_10_-KR8@ZIF-8 nanoparticles obtained on SEM. (C) Cell proliferation of BMDMs stimulated by gradient concentration of ZIF-8 and C_10_-KR8@ZIF-8 evaluated by CCK-8 kit on 24h, 48h and 72h. (D) Cell proliferation of BMSCs stimulated by gradient concentration of ZIF-8 and C_10_-KR8@ZIF-8 evaluated by CCK-8 kit on days1, 3, 5 and 7. (E) Live/dead staining images of BMSCs and RAW264.7 cells. (F) Representative data for the expression of BMDMs surface markers F4/80 determined by flow cytometry. The data are presented as the mean ± SD (n= 3). **p* < 0.05.


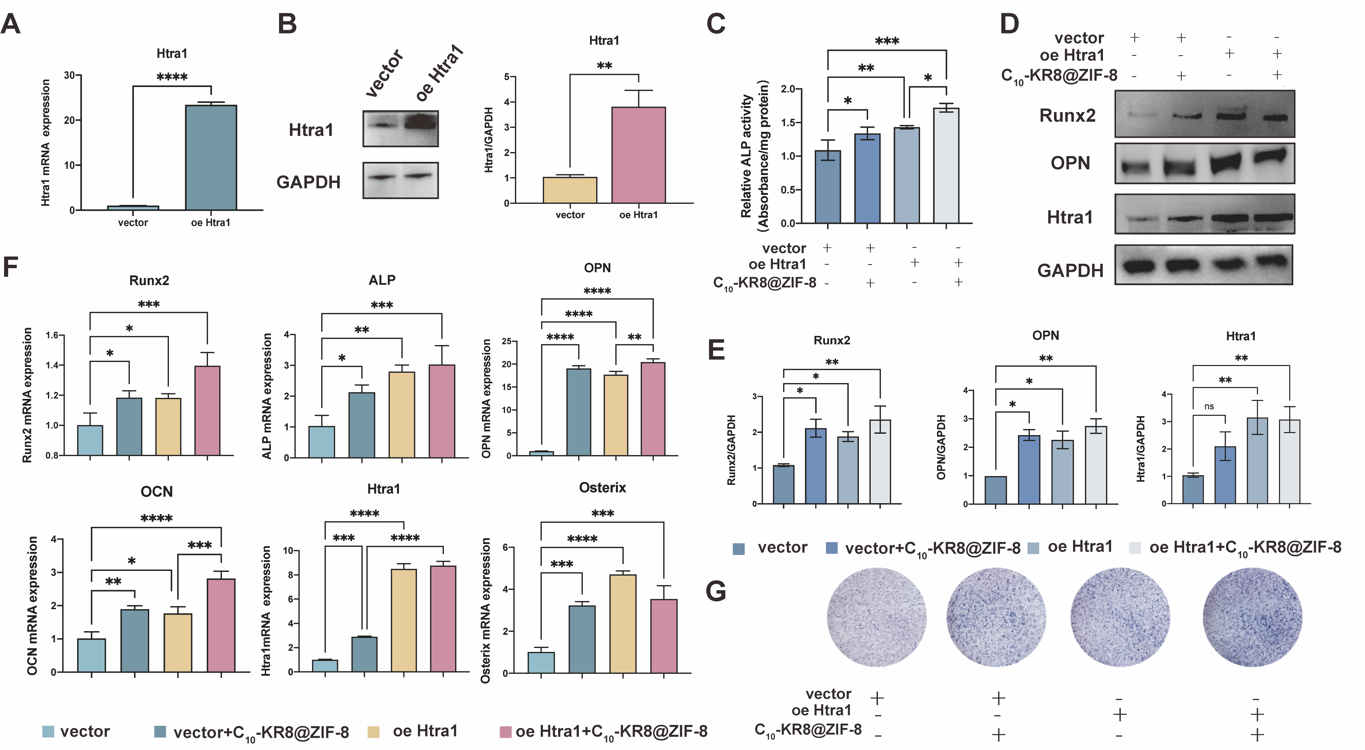


**Fig. S6.** Htra1 was critical for C_10_-KR8@ZIF-8–induced osteogenic differentiation of BMSCs in inflammatory condition. (A, B) Levels of Htra1 mRNA and protein expression in BMSCs infected with vector or oe-Htra1 plasmid. (C) Semiquantitative ALP activity of BMSCs infected with vector or the oe-Htra1 plasmid after C_10_-KR8@ZIF-8 treatment for 7 d in LPS-conditioned medium. (D) Western blot nalysis of Runx2, OPN and Htra1 expression in BMSCs infected with Vector or oe-Htra1 plasmid after C_10_-KR8@ZIF-8 treatment for 7 d in LPS-conditioned medium. (E) Quantitation of the protein expression levels of Runx2, OPN and Htra1. (F) qRT‒PCR analysis of Runx2, OCN, OPN, ALP, and Osterix mRNA in BMSCs infected with vector or the oe-Htra1 plasmid with or without C_10_-KR8@ZIF-8 for 7 d in LPS-conditioned medium. (G) ALP staining pictures of the BMSCs infected with vector or the oe-Htra1 plasmid after C_10_-KR8@ZIF-8 treatment for 7 d in LPS-conditioned medium. The data are presented as the mean ± SD (n= 3). ns: no significance; **p* < 0.05, ** *p* < 0.01 and *** *p* < 0.001.

**Table S1. The primer sequences used in qRT‒PCR analysis**

| Gene | Forward (5’-3’) | Reverse (3’-5’) |
| --- | --- | --- |
| IL-1β | GGGATTTTGTCGTTGCTTGT | CTGTGACTCGTGGGATGATG |
| IL-6 | TAGTCCTTCCTACCCCAATTTCC | TTGGTCCTTAGCCACTCCTTC |
| TNF-α | GCAACTGTTCCTGAACTCAACT | GCAACTGTTCCTGAACTCAACT |
| iNOS | GCGCTCTAGTGAAGCAAAGC | AGTGAAATCCGATGTGGCCT |
| IL-4 | CGGCAACTTTGTCCACGGA | TCTGTTACGGTCAACTCGGTG |
| IL-10 | GCAACTGTTCCTGAACTCAACT | GCAACTGTTCCTGAACTCAACT |
| TGF-β | CTCCAAGCCAAAGTCCTTAGAG | AGGAGCTGTCATTAGGGACATC |
| ALP | CACGGCGTCCATGAGCAGAAC | CAGGCACAGTGGTCAAGGTTGG |
| Runx2 | GCCTACTTACCCGTCTGACTTTGC | CCCTCCAGTTGCCCACTATTGC |
| OPN | GACGATGATGACGACGGATGAC | GTGTGCTGGCAGTGAAGGACTC |
| OCN | GGACCCTCTCTCTGCTCACTCTG | ACCTTACTGCCCTCCTGCTTGG |
| Osterix | ACCCCAAGATGTCTATAAGCCC | CGCTCTAGCTCCTGACAGTTG |
| COL1α1 | CCCTCCAGTTGCCCACTATTGC | GTCACCTTGTTCGCCTGTCTCAC |
| Htra1 | TCATCATCAGCATCAATGGACAGTC | TCAGGAACCACCGTAATCACAATG |
| GAPDH | ACAACTTTGGTATCGTGGAAGG | GCCATCACGCCACAGTTTC |
| Htra1siRNA | CGUCAUAAGUACAACUUUAtt | UAAAGUUGUACUUAUGACGtt |

**Table S2. Primary antibodies and dilutions used in this study**

| Name | Clone | Dilution | Supplier |
| --- | --- | --- | --- |
| IL-1β | polyclonal | 1:1000 | ABclonal |
| IL-6 | polyclonal | 1:1000 | Affinity |
| Arg-1 | polyclonal | 1:1000 | ABclonal |
| IL-10 | polyclonal | 1:1000 | Affinity |
| CD206 | polyclonal | 1:1000 | Affinity |
| CD86 | polyclonal | 1:1000 | Affinity |
| OPN | polyclonal | 1:1000 | Proteintech |
| Runx2 | monoclonal | 1:1000 | Abcam |
| HTRA1 | polyclonal | 1:500 | Proteintech |
| p-FAK | polyclonal | 1:1000 | Affinity |
| FAK | polyclonal | 1:1000 | Affinity |
| p-PI3K | polyclonal | 1:1000 | Affinity |
| PI3K | monoclonal | 1:1000 | Proteintech |
| p-AKT | monoclonal | 1:1000 | CST |
| AKT | monoclonal | 1:1000 | CST |
| p-YAP | monoclonal | 1:1000 | CST |
| YAP | polyclonal | 1:1000 | CST |
| GAPDH | monoclonal | 1:5000 | Proteintech |

**Table S3. Secondary antibodies and dilutions used in this study**

| Name | Dilution | Supplier |
| --- | --- | --- |
| Anti-mouse IgG HRP-linked Ab | 1:3000 | Abcam |
| Anti-rabbit IgG HRP-linked Ab | 1:3000 | Abcam |
| Anti-Rabbit lgG (Fluor488-conjugated) | 1:200 | Affinity |

**Table S4. The primer sequences used in qRT‒PCR analysis of rat maxillary bone tissues**

| Gene | Forward (5’-3’) | Reverse (3’-5’) |
| --- | --- | --- |
| Runx2 | GCTTCTCCAACCCACGAATG | GAACTGATAGGACGCTGACGA |
| OPN | GACGATGATGACGACGGATGAC | GTGTGCTGGCAGTGAAGGACTC |
| IL-1β | GGGATTTTGTCGTTGCTTGT | CTGTGACTCGTGGGATGATG |
| IL-6 | ACAGTGCATCATCGCTGTTC | CCGGAGAGGAGACTTCACAG |
| IL-4 | TGATGGGTCTCAGCCCCCACCTT | CTTTCAGTGTTGTGAGCGTGGACTC |
| TGF-β | TGAGTGGCTGTCTTTTGACG | CAGGAAGGGTCGGTTCATGT |
| GAPDH | TGGCCATACTTCTCCTCACC | TCTGTTCCGTCTGGGTTTTC |
